# Gut Microbiota and Immune Modulatory Properties of Human Breast Milk *Streptococcus salivarius* and *S. parasanguinis* Strains

**DOI:** 10.3389/fnut.2022.798403

**Published:** 2022-02-22

**Authors:** Shuo Li, Na Li, Chenwei Wang, Yi Zhao, Jie Cao, Xuejing Li, Ziyi Zhang, Yue Li, Xin Yang, Xiaoxin Wang, Chuanyan Che, Yufeng Zhao, Linghua Wang, Liping Zhao, Jian Shen

**Affiliations:** ^1^Key Laboratory of Systems Biomedicine (Ministry of Education), Shanghai Center for Systems Biomedicine, Shanghai Jiao Tong University, Shanghai, China; ^2^State Key Laboratory of Microbial Metabolism, School of Life Sciences and Biotechnology, Shanghai Jiao Tong University, Shanghai, China; ^3^Department of Animal Sciences, Anhui Science and Technology University, Chuzhou, China

**Keywords:** breast milk, *Streptococcus salivarius*, *Streptococcus parasanguinis*, gut microbiota, immunity

## Abstract

Human breast milk *Streptococcus* spp. are transferred to infant guts via breast feeding, but their effects on the gut microbiota and immunity remain unclear. In this study, we characterized gut microbiota and immune modulatory properties of human breast milk *S. salivarius* F286 and *S. parasanguinis* F278 that had been shown to be able to colonize gut. The two *Streptococcus* strains were orally administered to mouse pups individually at 1 × 10^7^ cells/day from postnatal Days 1 to 21. At postnatal week 3 (the weaning period), *S. salivarius* F286 reduced the colonic microbiota α-diversity, increased 21 amplicon sequence variants (ASVs), including bacteria from *Akkermansia, Intestinimonas*, and Lachnospiraceae, and decreased 52 ASVs, including bacteria from *Eubacterium, Bifidobacterium, Escherichia*-*Shigella*, and *Turicibacter*; however, *S. parasanguinis* F278 didn't change the colonic microbiota. Both *Streptococcus* strains reduced the ileal mRNA expression of cytokine/transcription factor representatives of T helper (Th) cells, including IFN-γ (Th1), Gata3 (Th2), and TGF-β (Treg) in 2-week-old suckling mice, and promoted the ileal expression of Foxp3 and TGF-β, which are representatives of anti-inflammatory Treg cells, in 3-week-old weaning mice. The two *Streptococcus* strains exhibited anti-inflammatory potential when incubated *in vitro* with human peripheral blood mononuclear cells and TNF-α-treated gut epithelial HT29 cells. In *C. elegans*, both strains activated immune response genes, which was associated with their lifespan-prolonging effects. Our results suggest that *S. salivarius* F286 and *S. parasanguinis* F278 may exert regulatory (anti-inflammatory) roles in gut immunity and *S. salivarius* F286 can modulate gut microbiota, and highlight the probiotic potential of milk *S. salivarius* and *S. parasanguinis* strains.

## Introduction

Human milk is an important inoculum for the pioneering commensal bacteria of the neonatal gut (1). It provides breastfed infants with 10^4^-10^8^ bacterial cells per day, which is estimated based on the average daily ingestion of 800 ml of breast milk by the infants (2). Bacterial culture-dependent studies have shown that *Streptococcus, Staphylococcus, Bifidobacterium, Lactobacillus*, and *Enterococcus* in maternal milk can colonize the infant gut (3, 4), and these results were confirmed by culture-independent studies in which microbial DNA of maternal milk and infant feces was sequenced (5, 6). Researchers have proposed that the milk microbiota is one of the milk components that influence the establishment of gut microbiota and the development of the gut immune system (7–9), but more studies are required to demonstrate the roles of human breast milk bacteria in modulating gut microbiota and educating immunity (10).

As reviewed (2, 7), researchers mainly previously investigated the antimicrobial and immunomodulatory properties of *Lactobacillus* and *Bifidobacterium* strains isolated from human breast milk. In contrast, studies on the effects of milk *Streptococcus* strains on gut microbiota and immunity are quite limited even though *Streptococcus* spp. are one of the most prevalent and abundant bacteria in the milk of mothers from different geographic locations (11, 12). Studies have reported only the *in vitro* antimicrobial capacity of breast milk *Streptococcus* (2); for instance, strains of *S. salivarius, S. parasanguinis* and *S. mitis* inhibited the growth of the pathogen *Staphylococcus aureus* (13), the *S. salivarius* VM18 strain inhibited type 1 human immunodeficiency virus (HIV-1) infection (14), and several *S. salivarius* strains agglutinated pathogens, such as *Staphylococcus aureus, S. pyogenes, Salmonella typhimurium, E. coli*, and *Shigella flexneri* (15). Fehr et al. found that *Streptococcus* that co-occurred in the milk of mothers and the stool of their infants affected the overall composition of infant gut microbiota, but the specific gut bacteria affected by the milk *Streptococcus* remained unknown (16). Therefore, it is necessary to study the capability of human breast milk *Streptococcus* strains to modulate gut microbiota and immunity (5, 17).

Previously, we established a human breast milk microbiota-associated mouse model by gavaging human breast milk to germfree mice and found that milk *S. salivarius* F286 and *S. parasanguinis* F278, in addition to *Staphylococcus lugdunensis* strains, predominated the gut of the recipient mice (18). This indicates that the two milk *Streptococcus* strains have great potential to colonize the human gut. Indeed, *S. parasanguinis* and *S. salivarius* were determined to be dominant bacteria in the gut of human infants in the first days of life (19), and *Streptococcus* spp. are dominant in the infant gut during the first 3 months of life (20).

Inoculating commensal bacteria into mice allows to study how bacteria influence the gut microbiota and host immunity *in vivo* (21–23). *In vitro* human peripheral blood mononuclear cells (PBMCs) and intestinal epithelial cells are widely used to evaluate the anti-/proinflammatory potential of bacteria in humans (24, 25). *C. elegans*, the immunity of which is closely connected with lifespan (26), is often utilized to screen for potential probiotic bacteria immune-modulatory properties of which contribute to lifespan-extension effects (27), and probiotic bacteria from *Lactobacillus, Bifidobacterium*, and *Propionibacterium* have been found to increase the longevity of *C. elegans* by activating immunity (28–30).

In the present study, we characterized the effects of human breast milk *S. salivarius* F286 and *S. parasanguinis* F278 and *Lactobacillus rhamnosus* GG (LGG), which was used as a benchmark strain, on gut microbiota composition in neonatal mice and their immunomodulatory capacities in neonatal mice, in *in vitro* human PBMCs and intestinal epithelial HT29 cells, and in *C. elegans*. *S. salivarius* F286 and *S. parasanguinis* F278 showed the anti-inflammatory and probiotic potential, and *S. salivarius* F286 modulated the gut microbiota composition of weaning mice. Our results provide insights into the biological functions of human breast milk *S. salivarius* and *S. parasanguinis*.

## Materials and Methods

### Bacterial Strains and Growth Conditions

*S*. *salivarius* F286 and *S. parasanguinis* F278 were isolated in our previous study in which human breast milk was gavaged to germ-free mice, and they were among the most abundant bacterial strains in the feces of the recipient mice; thus, *S*. *salivarius* F286 and *S. parasanguinis* F278 are milk bacteria that can colonize the gut (18). *L. rhamnosus* GG (LGG), which was used as a benchmark strain in the present study, was isolated from Culturelle® kids Packets (Culturelle, US) as described in the Supplementary Materials and Methods. *E. coli* OP50 was kindly provided by Prof. David Weinkove at Durham University.

*S. salivarius* F286 and *S. parasanguinis* F278 were cultured in M17 broth (Hopebiol, China) for 8 and 10 h, respectively, and LGG was cultured in MRS broth for 8 hours at 37°C in an anaerobic workstation to reach the growth plateau. The OD600 values for the plateau of *S. salivarius* F286, *S. parasanguinis* F278, and LGG were 0.62, 0.76, and 1.1, respectively. The concentrations of viable bacteria in the plateau culture were 1.63 × 10^9^, 1.06 × 10^9^, and 1.5 × 10^9^ colony forming units (CFU)/ml by serial dilution and plate counting for *S. salivarius* F286, *S. parasanguinis* F278, and LGG, respectively. *E. coli* OP50 was cultured aerobically in Luria-Bertani (LB) broth for 8 h at 37°C.

The details of the preparation of live bacterial cell suspensions for the mouse experiment, the bacterial culture supernatants and heat-killed bacterial cell suspensions for *in vitro* human cell experiments, and the live bacterial cell pellets for *C. elegans* experiments are described in the Supplementary Materials and Methods.

### Mouse Experiments

Specific pathogen-free (SPF) 7-week-old male C57BL/6J mice and 8-week-old female C57BL/6J mice were purchased from SLAC Inc. (Shanghai, China), kept at the animal facilities of Shanghai Jiao Tong University under a 12-h dark/light cycle at a temperature of 22°C ± 3°C, and fed normal chow (3.6 kcal/g, from Pu Lu Teng Biological Technology Inc., Shanghai, China) *ad libitum*. After two weeks of acclimatization, female mice were mated with male mice, and the pregnancy was validated and dated by visualizing the vaginal plug. Each cage housed 4 pregnant dams until gestation Day 16.5, and then they were separated into individual cages until delivery. Then, for the four dams that were originally housed in one cage, one dam was gavaged with 100 μl sterile PBS containing 10% skim milk every day as the placebo, and the other 3 dams were gavaged with 100 μl suspensions of individual strains of *S. salivarius* F286, *S. parasanguinis* F278, and LGG in PBS containing 10% skim milk at a dose of 1 × 10^8^ cells/day. From postnatal Day 1 to Day 21 (the age of 3 weeks), the pups of individual dams were orally fed 10 μl of the same bacterial suspension or the same placebo suspension that their mothers received before delivery by pipetting the suspension into the pups' mouth and holding the pups' nose for a few seconds intermittently until they swallowed the suspension, and the dose of individual bacterial strains was 1 × 10^7^ cells/day for each pup. Finally, four pup groups were generated and designated as the *S. salivarius* F286 (SsaF286) group, *S. parasanguinis* F278 (SpaF278) group, LGG group, and control (CTL) group.

The birth weight of each pup was measured at delivery. From birth to 3 weeks of age, the body weight was recorded for each pup once every 3 days. At the age of 2 weeks and 3 weeks, one male and one female of individual litters were randomly picked and sacrificed, and ileum fragments, colon tissue, and colon contents were collected. Details of the sampling procedures are provided in Supplementary Materials and Methods.

### Total Microbial DNA Extraction and Quantitative Real-Time PCR (qPCR) for *S. salivarius, S. parasanguinis* and LGG in the Colon Contents

Total microbial DNA was extracted as described in literature (31) from 129 colon content samples, including 61 and 68 samples from 2-week-old and 3-week-old pups, respectively.

*S. salivarius, S. parasanguinis*, and LGG in the pup colon contents were quantified by qPCR as described in Supplementary Materials and Methods. The sequences of the primers and their annealing temperatures are listed in Supplementary Table S1. Each PCR was performed in triplicate, and the amount of the targeted bacteria was expressed by the mean value of the three reactions as copies of targeted gene fragments per gram of colon content.

### 16S rRNA Gene V3-V4 Region Sequencing and Data Analysis

The sequencing library of the 16S rRNA gene V3-V4 region of 129 colon content samples was constructed according to the manufacturer's instructions (Part #15044223 Rev. B; Illumina Inc., USA) as previously described (32) and was sequenced on the MiSeq system (Illumina, Inc., USA) with the MiSeq reagent kit (600 cycles, catalog no. MS-102–3033; Illumina).

The raw paired-end reads were processed and amplicon sequence variants (ASVs) were generated with QIIME2 V2019.7. The sequence numbers of individual samples were downsized to 24,000. Principal coordinate analysis (PCoA) based on weighted UniFrac distances and permutational multivariate analysis of variance (PERMANOVA) were performed to provide the overview of gut microbiota composition dynamics in response to *S. salivarius, S. parasanguinis*, and LGG. Sparse partial least-squares discriminant analysis (sPLS-DA) models and Mann–Whitney *U* test were performed to identify ASVs that were changed by the three bacterial strains. Details of these analyses are described in Supplementary Materials and Methods.

### Histomorphology

Six-micrometer sections of the ileum and colon were stained with hematoxylin and eosin. Digital images of hematoxylin and eosin-stained sections were acquired with a Leica DMRBE microscope. Villus length was measured using Image-Pro Plus 6.0 software. For each mouse, the villus length was determined as the mean of at least 30 villi.

### RNA Isolation and RT–qPCR for the Mouse Experiment

Total RNA was extracted from the ileum with a RNeasy lipid tissue minikit (Qiagen, Germany). The transcript levels of cytokine and transcription factor representatives of Th1 (T-bet and IFN-γ), Th2 (Gata3 and IL-4), Th17 (Rorγt) and Treg (Foxp3 and TGF-β) cells, the anti-inflammatory cytokine IL-10, and antimicrobial peptides (Defβ1 and RegIIIγ) were determined by qPCR as described in Supplementary Materials and Methods. The sequences of the primers and their annealing temperatures are listed in Supplementary Table S1. The gene expression levels were determined using the ΔΔ*C*_*T*_ method (2^−Δ*ΔCT*^ method) with the β*-actin* gene as the reference gene.

### Bacterial Coculture With Human PBMCs

Freshly prepared PBMCs from 7 healthy human adults (1 female and 6 male) aged 21–40 were purchased from MT-Bio Company (Shanghai, China). The PBMCs were isolated using Ficoll-Paque density gradient centrifugation as described in Supplementary Materials and Methods.

Heat-killed bacterial cells were incubated with PBMCs at MOI of 1 (bacteria-to-cell ratio 1:1) and 10 (bacteria-to-cell ratio 10:1), and the bacterial culture supernatants were incubated with PBMCs at 2 and 10% final concentrations for 24 h at 37 °C in an atmosphere of air with 5% CO_2_. Each assay was performed in triplicate. IL-10 and IL-12p70 in PBMC culture supernatants were measured with ELISA. The detailed procedures were described in Supplementary Materials and Methods.

### Bacterial Coculture With TNF-α Stimulated HT29 Cells

Human gut epithelial HT29 cells were purchased from the Cell Bank of Type Culture Collection of the Chinese Academy of Sciences (Shanghai, China). HT29 cells stimulated with human recombinant 5 ng/ml TNF-α (PeproTech, USA) were incubated with heat-killed bacterial cells at MOI of 40 (bacteria-to-cell ratio 40:1) and 100 (bacteria-to-cell ratio 100:1), and with bacterial culture supernatants at final concentration of 10% for 6 h. Each assay was performed in triplicate. IL-8 in HT29 cell culture supernatants was measured with ELISA. The detailed procedures were described in Supplementary Materials and Methods.

### Lifespan Assays of *C. elegans* and Quantifying the Worm Gene Expression Levels

We used *C. elegans* strain SS104 glp-4 (bn2) for the lifespan experiments because it has a wild-type lifespan (33), is infertile at 25°C and fertile at 15°C. The SS104 worms were maintained on nematode growth medium (NGM) plates seeded with *E. coli* OP50 with standard techniques (34), and NGM was prepared as described previously (35). Synchronized SS104 *C. elegans* were cultured on NGM plates seeded with *E. coli* OP50 lawn at 15°C and then shifted to 25°C at the late L3 stage. L4 stage SS104 worms were transferred to plates of modified NGM (mNGM) that contained no peptone and were spread with 10 mg bacterial cells of individual strains of *S. salivarius* F286, *S. parasanguinis* F278, LGG, and *E. coli* OP50. For each lifespan assay, 100–150 worms per bacterial strain were allocated to five plates (20–30 worms/plate). The numbers of live and dead worms were counted every 48 h. A worm was considered dead when it did not move in response to the gentle touch of a platinum wire pick. On Days 11 after the SS104 worms were transferred to mNGM plates with lawn of individual bacterial strains, images of adult nematodes were taken using an IC80 HD camera (Leica, Germany), and the projection area of each worm was calculated as an index of body size using ImageJ software (30).

Five hundred worms fed individual bacterial strains from the L4 stage for 14 days were harvested, and total RNA was isolated. The transcript levels of immune genes were determined by qPCR with the 2^−ΔΔCT^ method with *act*-1 as the reference gene. The primer sequences are listed in Supplementary Table S2. The detailed procedures were described in Supplementary Materials and Methods.

### Statistical Analysis

**Figure 1:**
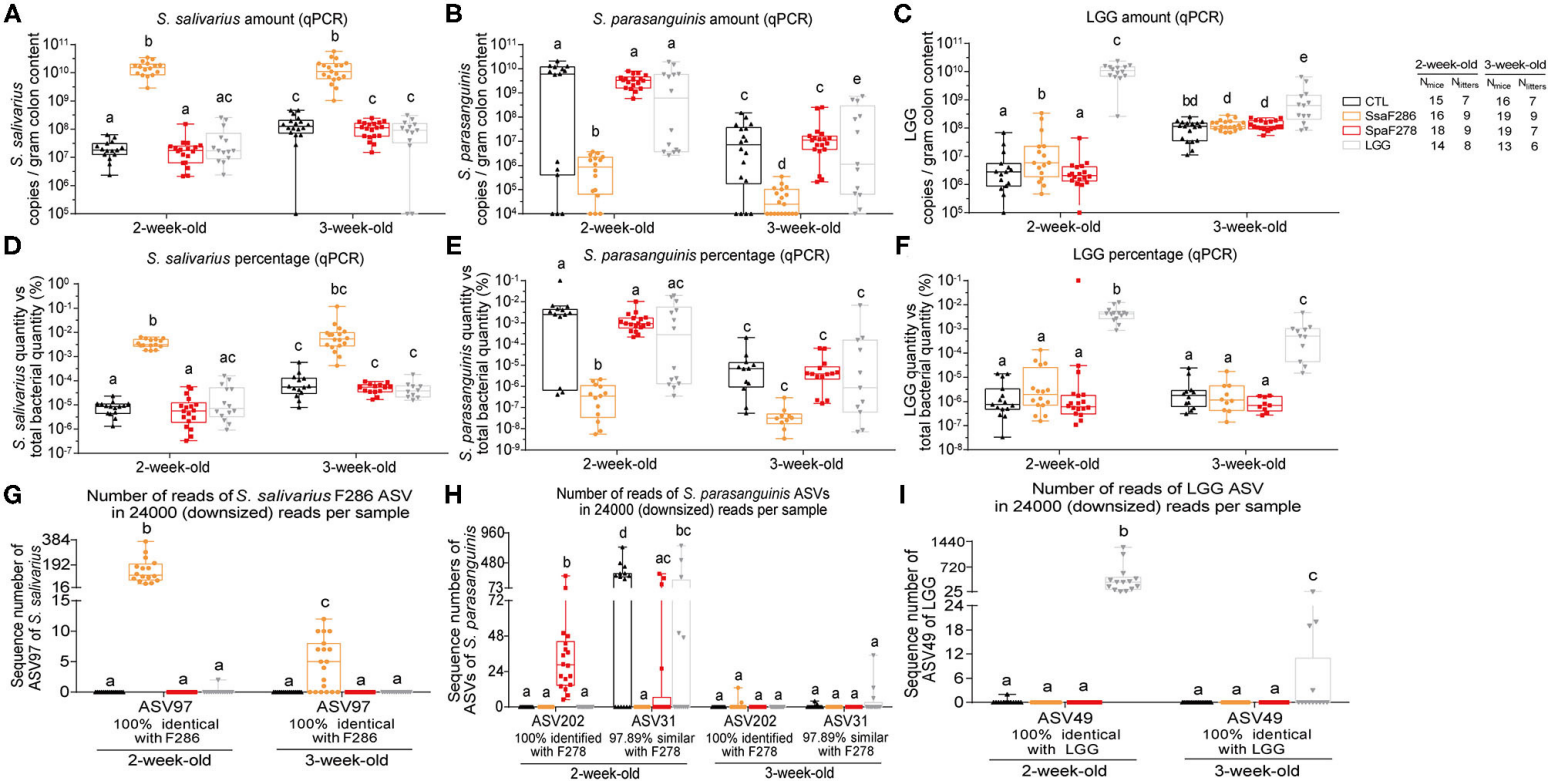
*S. salivarius* F286, *S. parasanguinis* F278 and LGG were recovered in the colon contents of mouse pups. **(A–C)** The amounts of *S. salivarius, S. paransanguinis* and LGG determined with qPCR as the copy numbers of the targeted gene of individual bacteria per gram of colon contents of 2- and 3-week-old mouse pups. **(D–F)** The percentage of copy numbers of the targeted genes of *S. salivarius, S. paransanguinis* and LGG accounted for the total copy numbers of the 16S rRNA gene (Supplementary Figure S1) in the colon contents of pups. **(G–I)** Sequencing read numbers of 16S rRNA gene V3-V4 region ASVs identified as *S. salivarius* F286, *S. paransanguinis* F278 and LGG in the colon contents of pups. In the box plots, the bottom and top are the 25th and 75th percentiles, respectively, and a line within the box marks the median. Whiskers above and below the box indicate the minimal and maximal values, respectively. Values of individual animal groups with different letters are considered significantly different by Mann–Whitney tests. CTL, Control group; SsaF286, *S. salivarius* F286 group; SpaF278, *S. parasanguinis* F278 group; LGG, LGG group.

**Figure 2:**
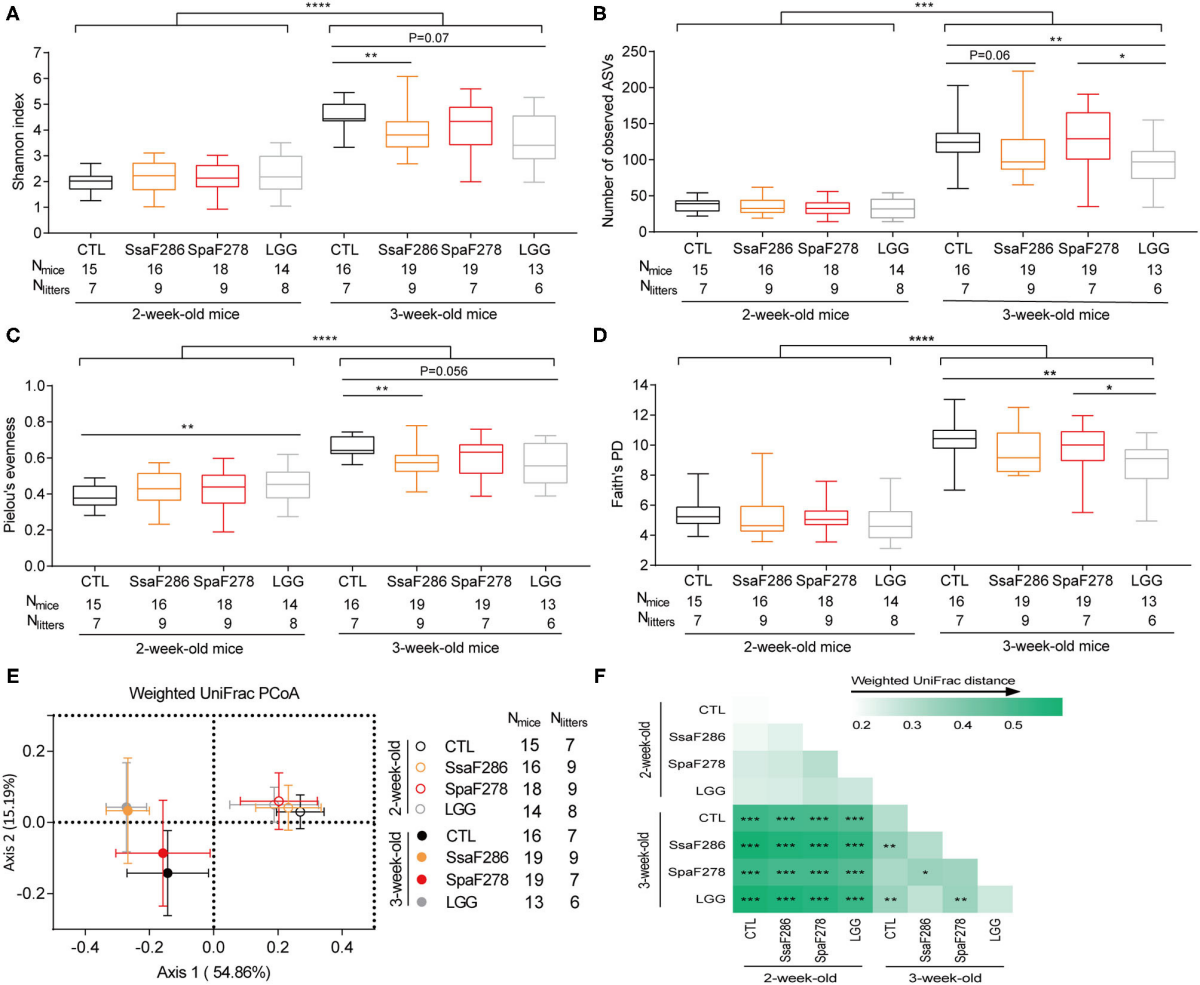
*S. salivarius* F286 and LGG reduced the α-diversity and changed the β-diversity of the colonic microbiota of 3-week-old pups. **(A)** Shannon index. **(B)** Number of observed ASVs. **(C)** Pielou's evenness index. **(D)** Phylogenetic diversity (PD) based on Faith's approach. In the box plot, the bottom and top are the 25th and 75th percentiles, respectively, and a line within the box marks the median. Whiskers above and below the box indicate min to max. The values of different animal groups were compared with the Mann–Whitney test. *p < 0.05; **p < 0.01; ***p < 0.001; ****p < 0.0001. **(E)** PCoA plot based on weighted UniFrac distance of colonic microbiota of 2- and 3-week-old pups of different groups. **(F)** Heatmap of the weighted UniFrac distances between each two groups and permutational multivariate analysis of variance (PERMANOVA; 9,999 permutations). *p < 0.05; **p < 0.01; ***p < 0.001. CTL, Control group; SsaF286, *S. salivarius* F286 group; SpaF278, *S. parasanguinis* F278 group; LGG, LGG group.

**Figure 3:**
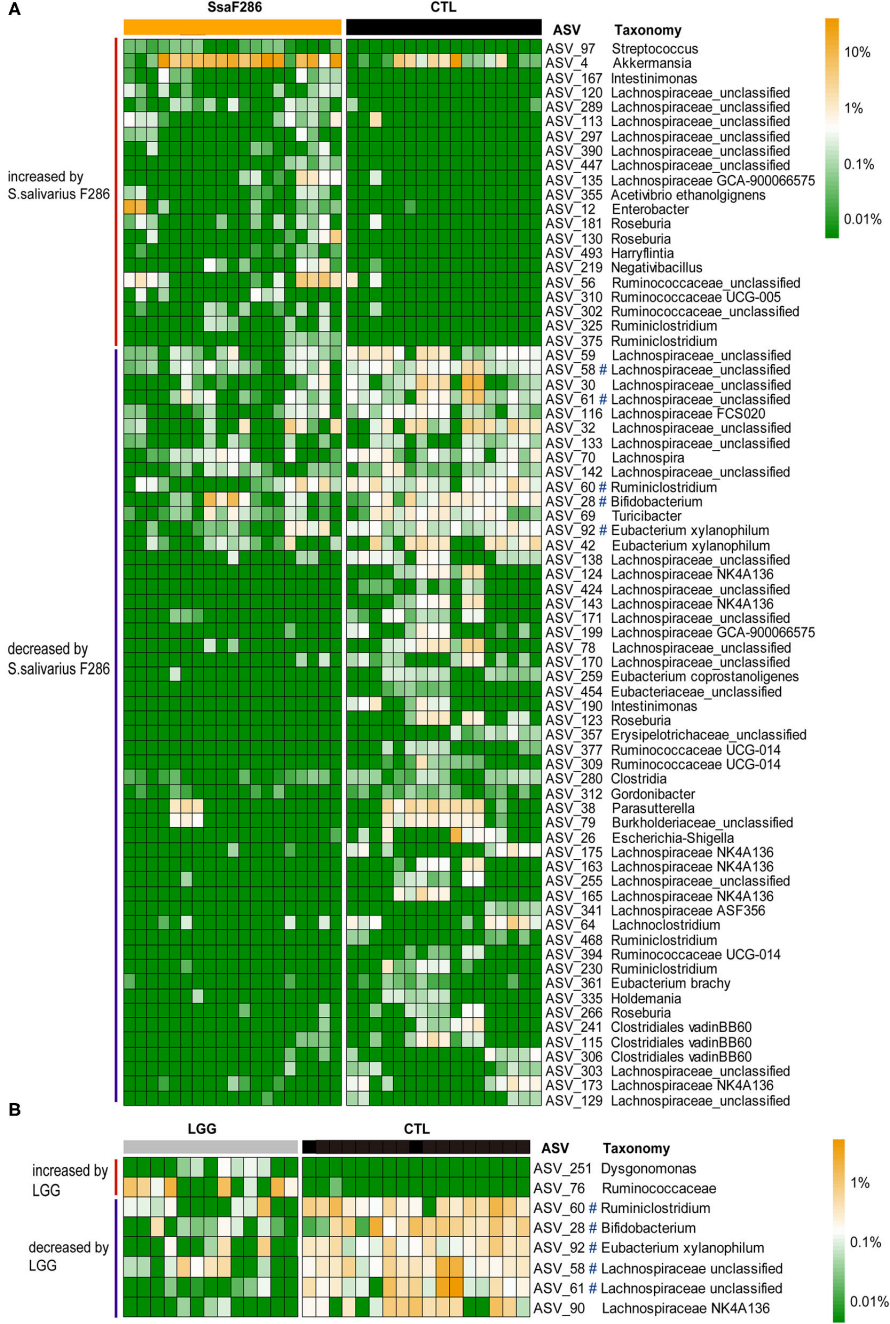
ASVs changed by *S. salivarius* F286 and LGG at the age of 3 weeks. **(A)** Heatmap of the normalized and log_10_-transformed relative abundance of 73 ASVs changed by *S. salivarius* F286 in each sample. **(B)** Heatmap of the normalized and log_10_-transformed relative abundance of 8 ASVs changed by LGG in each sample. The taxonomy of the ASVs is shown. ^#^ASVs decreased by both *S. salivarius* F286 and LGG. CTL, Control group; SsaF286, *S. salivarius* F286 group; LGG, LGG group.

**Figure 4:**
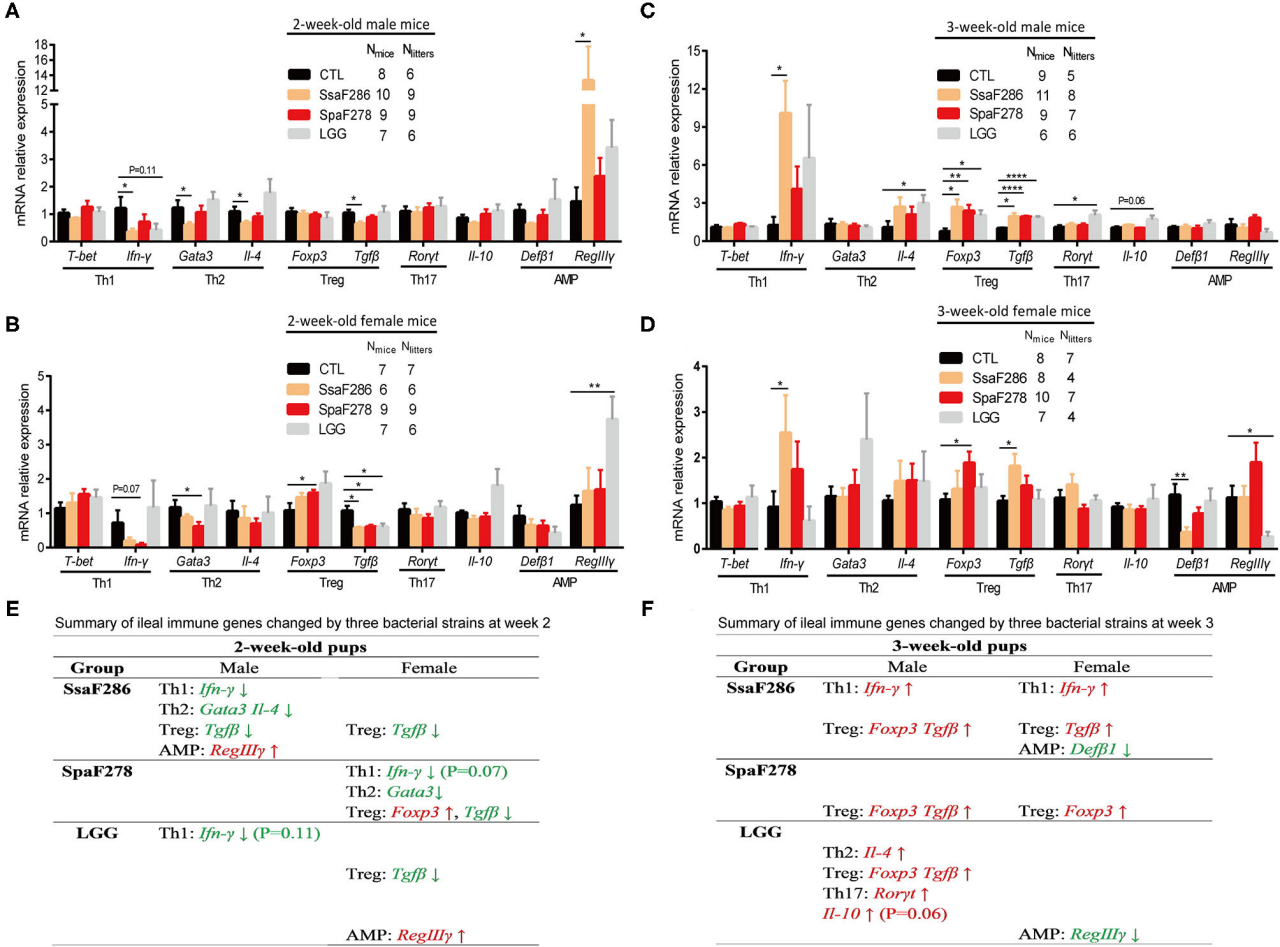
*S. salivarius* F286, *S. parasanguinis* F278 and LGG modulated the ileal expression of immune genes in 2- and 3-week-old mouse pups. **(A–D)** mRNA expression levels of ileal transcription factors and cytokines representative of four Th cell lineages and antimicrobial peptides in 2-week-old male **(A)** and female **(B)** pups and in 3-week-old male **(C)** and female **(D)** pups. All mRNA quantification data were normalized to the housekeeping gene β*-actin*. Gene expression levels were expressed as values relative to the control (CTL) group. Data are presented as the mean ± s.e.m. The data of *S. salivarius* F286-, *S. parasanguinis* F278- and LGG-fed animals were compared to those of control animals with Student's t test. *p < 0.05; **p < 0.01; ****p < 0.0001. **(E,F)** Summary of ileal immune genes that tended to be (*p* values provided in the parentheses following the gene) or were significantly changed by individual bacterial strains at the age of 2 weeks **(E)** and 3 weeks **(F)**. Genes in green and red are decreased and increased, respectively, by the bacteria. AMP, antimicrobial peptides; CTL, Control group; SsaF286, *S. salivarius* F286 group; SpaF278, *S. parasanguinis* F278 group; LGG, LGG group.

**Figure 5:**
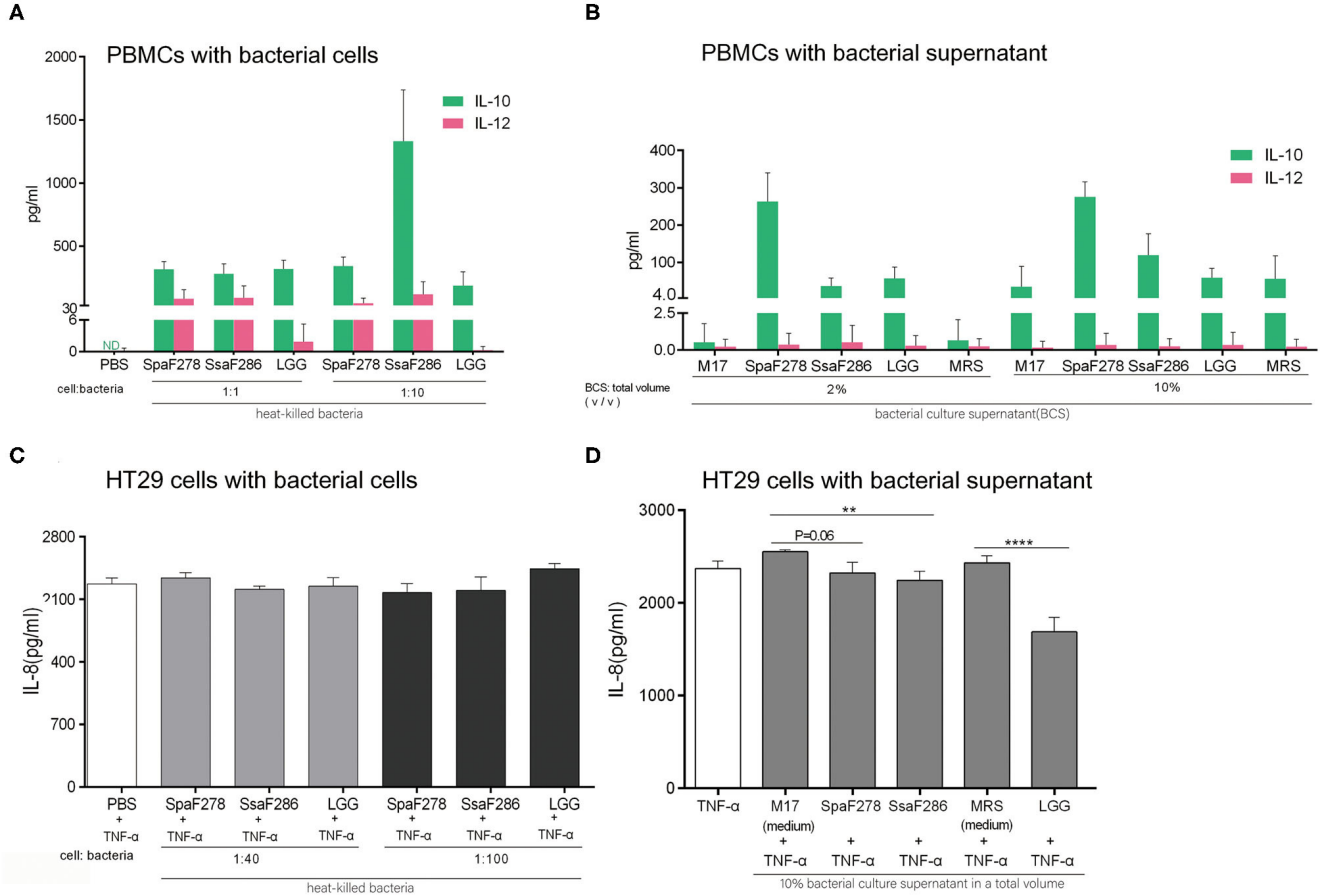
*S. salivarius* F286, *S. parasanguinis* F278 and LGG exhibited anti-inflammatory capacity in *in vitro* assays with human PBMCs and TNF-α-treated gut epithelial HT29 cells. **(A,B)** Anti-inflammatory IL-10 and pro-inflammatory IL-12 production by PBMCs stimulated with heat-killed bacterial cells **(A)** and culture supernatants **(B)** of individual bacterial strains for 24 h. Data are expressed as the mean ± SD of the results of the assays of 5 and 7 healthy blood donors for the bacterial cells and supernatants, respectively. **(C,D)** The effects of heat-killed bacterial cells **(C)** and culture supernatants **(D)** of individual bacterial strains on IL-8 production by HT29 cells treated with 5 ng/ml TNF-α. Data are represented as the mean ± SD of at least triplicate measurements, and differences were compared with Student's *t-*test. **p < 0.01; ****p < 0.0001. PBS, phosphate buffered saline. M17, the unfermented sterile bacterial growth medium for *S. salivarius* F286 and *S. parasanguinis* F278. MRS, the unfermented sterile bacterial growth medium for LGG.

**Figure 6:**
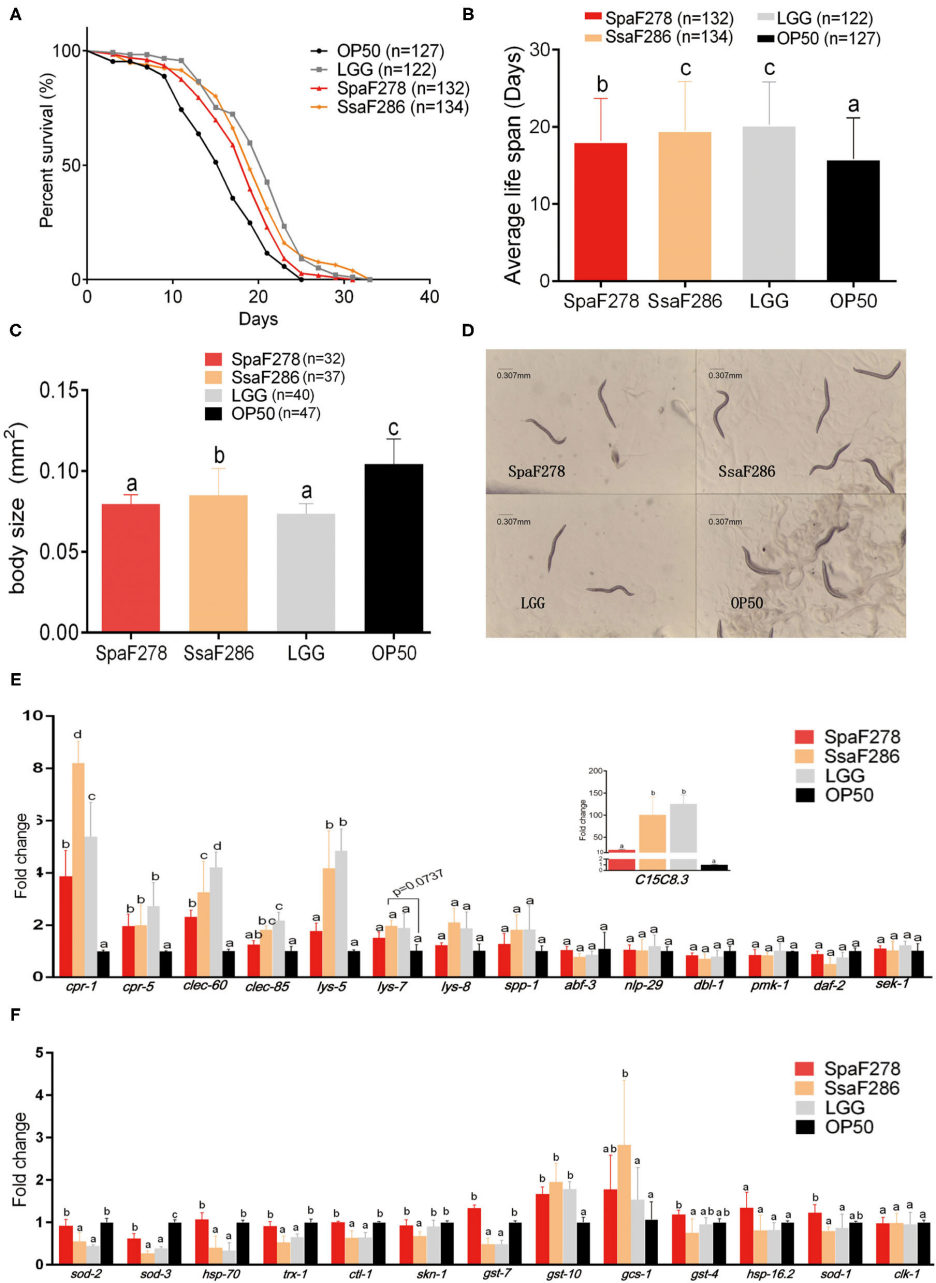
Effects of *S. salivarius* F286, *S. parasanguinis* F278 and LGG cells on the lifespan, body size and expression of immune and antioxidant genes of *C. elegans*. **(A,B)** Survival curves **(A)** and average lifespan **(B)** of *C. elegans* fed *E. coli* OP50 (standard food), *S. parasanguinis* F278, *S. salivarius* F286, and LGG. Data are shown as the mean ± SD, and animal groups with different letters are significantly different by the log-rank test. **(C,D)** Body size **(C)** and microscopic images **(D)** of *C. elegans* 11 days after being fed individual bacterial strains. All images were acquired at the same magnification, and a scale bar of 0.307 mm is indicated. **(E,F)** Expression levels of immune genes **(E)** and antioxidative genes **(F)** in *C. elegans* 14 days after being fed individual bacterial strains. All mRNA quantification data were normalized to the housekeeping gene *act-1*. The gene expression levels were expressed as values relative to the *E. coli* OP50 group. Data are shown as the mean ± SD. For the individual genes, the animal groups with different letters are significantly different by Student's *t*-test. OP50, *E. coli* OP50 group; SsaF286, *S. salivarius* F286 group; SpaF278, *S. parasanguinis* F278 group; LGG, LGG group.

The Mann–Whitney *U* test was used to compare the difference in the bacterial amounts determined with qPCR, α-diversity indices and bacterial ASV amounts (Figures 1–**3**, Supplementary Figures S1, S5, S7, S8, S10, S11) because these data did not obey a normal distribution. Student's *t*-test was used to determine the statistical significance of the mouse gene expression levels, the amounts of cytokines secreted by human PBMCs and HT29 cells, and *C. elegans* body size and gene expression levels (**Figures 4**–**6**, Supplementary Figures S6, S12). The Pearson's correlation test was used to examine the correlation between the abundances of *S. parasanguinis* ASVs and *S. parasanguinis* amounts determined with qPCR (Supplementary Figures S3). The Mann–Whitney *U-*test, Student's *t*-test, and Pearson's correlation were performed using GraphPad Prism 6 software (GraphPad Software, USA). PERMANOVA was performed in the “vegan” R package (Figure 2, Supplementary Figure S9). The *C. elegans* survival curves were plotted with the Kaplan–Meier method, and the lifespan differences between groups were compared with the log-rank test on the OASIS2 platform (Online Application for Survival Analysis 2, https://sbi.postech.ac.kr/oasis2/) (36). Differences were considered significant when the *P* value was < 0.05.

## Results

### The Two Breast Milk *Streptococcus* Strains and LGG Were Recovered in the Colons of Mouse Pups

Newborn mouse pups were divided into 4 groups with 7–9 litters/group. One group was the control (CTL) group that was orally administered phosphate-buffered saline (PBS) containing 10% skimmed milk as a placebo, and the three other groups were orally administered individual strains of *S. salivarius* F286, *S. parasanguinis* F278, and LGG that were suspended in PBS containing 10% skimmed milk at a dose of 1 × 10^7^ cells/day from postnatal Day 1 to 21 (age of 3 weeks).

At 2 and 3 weeks of age, using quantitative PCR (qPCR), we determined the abundance of *S. salivarius* and *S. parasanguinis*, the bacterial species that the strains *S. salivarius* F286 and *S. parasanguinis* F278 belong to, respectively, and LGG in the colon contents of pups as the copies of target gene per gram of colon content (Figures 1A–C) and as percentages of the target gene copies relative to total 16S rRNA gene copies (Figures 1D–F, Supplementary Figure S1). The colonic levels of *S. salivarius* and LGG at the two ages significantly increased upon daily oral feeding of *S. salivarius* F286 and LGG (Figures 1A,C,D,F). The median *S. salivarius* amounts in *S. salivarius* F286-fed pups were 1.5 × 10^10^ and 1.1 × 10^10^ copies of target gene/g colon content at age 2 and 3 weeks, respectively, which were over 100-fold higher than those of other three animal groups not receiving *S. salivarius* F286 (Figure 1A). The median LGG levels in LGG-fed pups reached 1.1 × 10^10^ and 6.3 × 10^8^ copies of target gene/g colon content at weeks 2 and 3, respectively, which were approximately 1000-fold and 6-fold higher than those of the three groups of pups without LGG administration at week 2 and 3, respectively (Figure 1C). *S. parasanguinis* F278-fed and control pups showed no significant difference in the colonic *S. parasanguinis* levels at week 2 (median 3.3 × 10^9^ copies of target gene/g colon content for the former and 6.1 × 10^9^ for the latter) and week 3 (median 1.1 × 10^7^ copies of target gene/g colon content for *S. parasanguinis* F278-fed pups and 7.2 × 10^6^ for control pups) (Figure 1B), indicating that pups were likely to have endogenous *S. parasanguinis* strain(s) other than *S. parasanguinis* F278 in the colon.

Amplicon sequence variants (ASVs) of 16S rRNA gene sequences of microbial communities can differentiate different strains/ecotypes within a bacterial species (37). To check whether *S. parasanguinis* F278 feeding elevated the abundance of *S. parasanguinis* F278 but not the endogenous *S. parasanguinis* strain(s) in the colon, we sequenced the 16S rRNA gene V3-V4 region of the colon contents of four groups of pups at age 2 weeks (61 pups) and 3 weeks (68 pups). A total of 4,733,087 high-quality reads (35,856 ± 5,394 reads per sample) were obtained, and 888 ASVs were generated. The sequences of all samples were downsized to 24,000 to standardize the sequencing depth.

Two ASVs, ASV202 and ASV31, showed 100% similarity with known *S. parasanguinis* isolates (Supplementary Table S3), and were unambiguously clustered with *S. parasanguinis* in the phylogenetic tree consisting of 16S rRNA gene sequences of *Streptococcus* spp. (Supplementary Figure S2). Besides, in all 2-week-old mice, the sum of sequence numbers of the two ASVs was significantly correlated with the *S. parasanguinis* quantity determined as copies of *groEL* gene per gram of colon content using qPCR (Pearson correlation *r* = 0.69, *p* < 0.0001, Supplementary Figures S3A,B, Supplementary Table S4); in mice in which only ASV202 or ASV31 was detected, the sequence number of the detected ASV was significantly correlated with *S. parasanguinis* amounts quantified with qPCR (Pearson correlation for ASV202 r = 0.62, *p* = 0.02; Pearson correlation for ASV31 r = 0.51, *p* = 0.045; Supplementary Figures S3C,D, Supplementary Table S4). These results suggested that ASV202 and ASV31 represented *S. parasanguinis* in the pups.

ASV202, which was 100% identical to the 16S rRNA gene of *S. parasanguinis* F278 (Supplementary Table S3), clustered most closely to *S. parasanguinis* F278 in the phylogenetic tree consisting the 16S rRNA gene sequences of 115 *S. parasanguinis* strains (Supplementary Figure S4), and was detected in all *S. parasanguinis* F278-fed pups but in none of the pups of the three other groups that did not receive *S. parasanguinis* F278 at the age of 2 weeks (Figure 1H, Supplementary Tables S3, S4). These results suggest that *S. parasanguinis* F278 was represented by ASV202 and arrived at the colon of the pups.

ASV31, which was 100% identical to the *S. parasanguinis* KCOM strain but only 97.89% similar to *S. parasanguinis* F278 (Supplementary Table S3), distributed in a different cluster of *S. parasanguinis* strains from ASV202 (Supplementary Figure S4), and did not appear simultaneously with ASV202 in 30 out of 34 mice in which either of the two ASVs was detected (Supplementary Table S4); in addition, ASV31 was present in most (9 out of 15) control pups and half (7 out of 14) of LGG-fed pups, but in only 4 out of 18 *S. parasanguinis* F278-fed pup (Supplementary Table S3), therefore, ASV31 was very likely to represent the endogenous *S. parasanguinis* strain other than *S. parasanguinis* F278. The amounts of ASV31 in *S. parasanguinis* F278-fed pups were significantly lower than those in control pups at the age of 2 weeks (Figure 1H, Supplementary Table S3), indicating that *S. parasanguinis* F278 feeding decreased the endogenous *S. parasanguinis* strain.

At the age of 3 weeks, as the percentages of *S. parasanguinis* in the colon of the four groups of pups were less than 10^−5^% according to qPCR (Figure 1E), the *S. parasanguinis* sequences were hardly detected at a sequencing depth of 24,000 reads/sample (Figure 1H). However, it is very likely that colonic *S. parasanguinis* F278 was still elevated by its oral administration at 3 weeks, because *S. parasanguinis* F278 feeding could decrease the endogenous *S. parasanguinis* strain in *S. parasanguinis* F278-fed pups as shown by 16S rRNA gene ASV analysis for week 2 (Figure 1H), but the colonic *S. parasanguinis* levels of 3-week-old *S. parasanguinis* F278-fed pups were as high as the levels of the endogenous *S. parasanguinis* strain in the control pups according to the qPCR results (Figures 1B,E).

Consistent with the qPCR results, *S. salivarius* F286- and LGG feeding significantly enhanced the colonic amount of ASV sequence of which was 100% identical to that of *S. salivarius* F286 and LGG, respectively (Figures 1G,I).

The male and female pups did not show differences in the abundance of *S. salivarius* F286, *S. parasanguinis* F278 and LGG at the ages of 2 and 3 weeks (Supplementary Figure S5).

The qPCR and 16S rRNA gene ASV results showed that *S. salivarius* F286, *S. parasanguinis* F278 and LGG was present in the colon of the mouse pups.

### The Two Breast Milk *Streptococcus* Strains and LGG Did Not Affect the Pups' Body Weight or Intestinal Villus Length

We measured the pup birth weight and body weight every 3 days until they were 3 weeks old. The body weight (Supplementary Figure S6A) and relative body weight, which was calculated as the fold change of body weight relative to birth weight (Supplementary Figure S6B), of *S. salivarius* F286-, *S. parasanguinis* F278-, and LGG-fed pups were not different from those of the control pups. *S. salivarius* F286-, *S. parasanguinis* F278-, and LGG-feeding did not change the villus length of the jejunums, ileums, and colons of the pups at age 2 and 3 weeks compared to those of the control (Supplementary Figure S7), suggesting that *S. salivarius* F286 and *S. parasanguinis* F278 did not impair the development of intestinal villus in neonatal mice.

These results indicate that daily oral feeding of *S. salivarius* F286, *S. parasanguinis* F278, and LGG at a dose of 1 × 10^7^ CFU/day in the first 3 weeks of life was safe for neonatal mice.

### *S. salivarius* F286 and LGG Changed the Colonic Microbiota Diversity of Mouse Pups at the Age of 3 Weeks

The 16S rRNA gene V3-V4 region sequencing data of the pups' colon contents at age 2 and 3 weeks were analyzed to study the effects of *S. salivarius* F286, *S. parasanguinis* F278, and LGG ingestion on the diversity of early life gut microbiota. Because the male and female pups did not display differences in the α- and β-diversity of the gut microbiota at ages 2 and 3 weeks (Supplementary Figure S8, Supplementary Table S5), the sequencing data of the two sexes were analyzed together.

Regardless of the animal groups, 2-week-old pups, food of which was mainly dam milk, showed significantly lower α-diversity indices (Shannon index, number of observed ASVs, Faith's phylogenetic diversity, and Pielou's evenness index) than those of the 3-week-old pups that started to nibble a few solid chows (Figures 2A–D). This is in accordance with previous reports that breastfeeding is associated with lower α-diversity and that intake of solid food increases the α-diversity of gut microbiota in infants (38, 39).

At 2 weeks, compared to the control, the two milk *Streptococcus* strains and LGG did not significantly change the α-diversity (Figures 2A–D) except that LGG slightly increased Pielou's evenness (Figure 2C). At 3 weeks, both *S. salivarius* F286 and LGG significantly decreased the α-diversity of the pups' colonic microbiota compared to that of the control animals. *S. salivarius* F286 significantly reduced the Shannon index (Figure 2A) and Pielou's evenness (Figure 2C) and tended (*p* = 0.06) to reduce the number of observed ASVs (Figure 2B); LGG significantly lowered the number of observed ASVs (Figure 2B) and Faith's PD (Figure 2D) and tended to decrease the Shannon index (*p* = 0.07, Figure 2A) and Pielou's evenness (*p* = 0.056, Figure 2C). *S. parasanguinis* F278 did not change the α-diversity of the pups' colonic microbiota compared to that of the control pups at week 3 (Figures 2A–D), which is in concordance with the result that *S. parasanguinis* F278 did not enhance the colonic level of *S. parasanguinis* due to the presence of the endogenous *S. parasanguinis* strain in the control animals (Figures 1B,E,H).

Weighted UniFrac PCoA based on the ASV abundance matrix was performed to visualize the gut microbiota structure of the four pup groups at ages 2 and 3 weeks (Figure 2E), and PERMANOVA for PCoA was used to test whether the difference in the gut microbiota composition was statistically significant among the animal groups (Figure 2F). Regardless of the animal group, the gut microbiota composition was significantly different between 2-week-old and 3-week-old pups (Figures 2E,F), which reflects the diet shift of the young mice from pure maternal milk to milk supplemented with chow. At week 2, there was no difference in gut microbiota structure among the control, *S. salivarius* F286, *S. parasanguinis* F278, and LGG animals (Figures 2E,F, Supplementary Figures S9A–C). At week 3, *S. salivarius* F286 and LGG, but not *S. parasanguinis* F278, significantly changed the pups' gut microbiota structure compared to that of the control animals (Figures 2E,F, Supplementary Figures S9D–F).

sPLS-DA models were constructed for the *S. salivarius* F286-fed and control pups (Supplementary Figures S10A,B) and for the LGG-fed and control pups (Supplementary Figures S11A,B) at the age of 3 weeks to identify the feature ASVs contributing to the differentiation of colonic microbiota of the *S. salivarius* F286 group and LGG group from that of the control pups, and then Mann–Whitney tests were performed to compare the abundances of individual feature ASVs between the *S. salivarius* F286-/LGG-treated and control pups (Supplementary Figures S10C, S11C). ASVs, abundances of which both contributed to microbiota composition differentiation in the sPLS-DA models and showed a significant difference between the two animal groups in the Mann–Whitney *U*-test, were considered to be bacteria that were changed by *S. salivarius* F286 or LGG. *S. salivarius* F286 increased the abundance of 21 ASVs, including bacteria belonging to *Streptococcus, Akkermansia, Intestinimonas*, Lachnospiraceae, and Ruminococcaceae, and decreased 52 ASVs, including bacteria from *Eubacterium, Bifidobacterium, Ruminiclostridium, Escherichia*-*Shigella, Turicibacter*, and Lachnospiraceae (Figure 3A). LGG enriched 2 ASVs from unclassified Ruminococcaceae and *Dysgonomonas* and reduced 6 ASVs that were representative of *Ruminiclostridium, Bifidobacterium, Eubacterium*, and Lachnospiraceae (Figure 3B). Interestingly, 5 out of the 6 LGG-reduced ASVs were also decreased by *S. salivarius* F286 (Figure 3).

### The Two Breast Milk *Streptococcus* Strains and LGG Modulated the Ileum Immune Gene Expression in Mouse Pups

Because the balance among ileal Th cells maintains the homeostasis of mucosal immunity (40), we performed qPCR to quantify the mRNA expression levels of transcription factor and cytokine genes that are important for the differentiation and function of proinflammatory Th1, Th2 and Th17 cells and anti-inflammatory Treg cells in the ileum of pups at 2 and 3 weeks of age (Figure 4) (41). qPCT was also performed to quantify the expression levels of antimicrobial peptide (AMP) β-defensin 1 (Defβ1) and regenerating islet-derived protein 3 gamma (RegIIIγ), which are parts of the innate immune response (41) (Figure 4). Because the expression levels of immune genes showed significant differences between sexes at two time points (Supplementary Figure S12), the gene expression levels of bacteria-treated pups were compared to those of their control counterparts in each sex (Figure 4).

At 2 weeks, *S. salivarius* F286 significantly downregulated the expression of multiple transcription factor/cytokine representatives of Th cell lineages in male pups, i.e., IFN*-*γ (the cytokine representative of Th1 cells), Gata3 and IL-4 (the transcription factor and cytokine of Th2 cells), and TGF-β (Treg cell cytokine), and decreased Treg cell-related TGF-β expression in female pups, and it remarkably increased the expression of AMP RegIIIγ in male pups (Figures 4A,B,E). *S. parasanguinis* F278 did not change the ileal expression of the immune genes of interest in 2-week-old male pups, but it tended to (*p* = 0.07) reduce the expression of Th1 cell-associated IFN-γ and significantly reduced expression of Th2 cell-associated Gata3 and Treg cell-related TGF-β in 2-week-old female pups (Figures 4A,B,E). LGG tended to (*p* = 0.11) downregulate the expression of Th1 cell-associated IFN-γ in male pups, significantly decreased Treg cell-related TGF-β expression, and significantly upregulated AMP RegIIIγ expression in female pups (Figures 4A,B,E). In summary, *S. salivarius* F286, *S. parasanguinis* F278 and LGG showed suppressive effects on ileal Th cells of 2-week-old pups, and *S. salivarius* F286 and *S. parasanguinis* F278 did so mainly in males and females, respectively.

At 3 weeks, *S. salivarius* F286 significantly upregulated the expression of the Th1 cell cytokine IFN-γ and the Treg cell cytokine TGF-β in both female and male pups, and it increased the expression of the Treg cell marker Foxp3 in male pups and decreased AMP Defb1 expression in female pups (Figures 4C,D,F). *S. parasanguinis* F278 enhanced the expression of the Treg cell marker Foxp3 in 3-week-old pups of both sexes and increased the expression of the Treg cell cytokine representative TGF-β in 3-week-old male pups (Figures 4C,D,F). LGG increased the expression of the Th2 cell cytokine IL-4, Th17 cell-related Rorγt, Treg cell-associated TGF-β and Foxp3, and the anti-inflammatory cytokine IL-10 in 3-week-old male pups and downregulated the expression of the AMP RegIIIγ in 3-week-old female pups (Figures 4C,D,F). *S. salivarius* F286, *S. parasanguinis* F278 and LGG all enhanced the expression of anti-inflammatory Treg cell-related genes in 3-week-old pups, and *S. salivarius* F286 and *S. parasanguinis* F278 did so in both genders.

### The Two Breast Milk *Streptococcus* Strains and LGG Showed Anti-inflammatory Capability When Incubated *in vitro* With Human PBMCs and Gut Epithelial HT29 Cells

Because the two milk *Streptococcus* strains and LGG promoted the expression of intestinal anti-inflammatory Treg cell genes in weaning mice, we then evaluated the anti-inflammatory potential of the two milk *Streptococcus* strains and LGG in *in vitro* human PBMC and human gut epithelial HT29 assays. Human PBMCs and HT29 cells were used because the anti-inflammatory cytokine IL-10 and pro-inflammatory IL-12 release patterns of human PBMCs in response to bacteria and the effects of the bacteria on the chemoattractant cytokine IL-8 secretion of TNF-α-treated human gut epithelial HT29 cells reflect the anti-inflammatory potential of bacteria in humans (24, 42). As live *S. salivarius* F286 and *S. parasanguinis* F278 grew in human cell culture in our preliminary experiments, we tested the heat-killed bacterial cells and culture supernatants of the two milk *Streptococcus* strains and LGG in the PBMC and HT29 cell assays as other researchers did when they studied human commensal and infection-associated *Streptococcus* strains from the oral cavity and blood (24, 43).

At multiplicity of infection (MOI) of 1 and 10 bacteria per PBMC, the heat-killed cells of *S. salivarius* F286 and *S. parasanguinis* F278 induced PBMCs to secrete more IL-10 relative to IL-12, and LGG cells induced the secretion of major IL-10 and little IL-12 (Figure 5A). At concentrations of 2% and 10%, the culture supernatants of the three strains induced the production of high levels of IL-10 and low levels of IL-12 (Figure 5B).

At MOI of 40 and 100, the heat-killed cells of the three bacterial strains neither promoted nor decreased the TNF-α-induced IL-8 secretion of HT29 cells (Figure 5C). At a concentration of 10%, the culture supernatants of the three bacterial strains all reduced IL-8 secretion compared to unfermented sterile bacterial growth media (M17 for two *Streptococcus* strains and MRS for LGG) (Figure 5D).

In summary, the two breast milk *Streptococcus* strains and LGG exhibited anti-inflammatory potential in the human PBMC and HT29 cell assays, which is consistent with their promotive effects on the expression of intestinal anti-inflammatory Treg cell genes in weaning mice.

### The Cells of Two Breast Milk *Streptococcus* Strains and LGG Prolonged the Lifespan of *C. elegans*

Because *C. elegans*, a model experimental animal, has a short lifespan and its immunity is closely connected with the lifespan (26), it is used in the laboratory to screen for probiotic bacteria whose immune-modulatory properties contribute to lifespan extension effects (27–30). Therefore, we studied the probiotic potential and immune-modulation effects of the two breast milk *Streptococcus* strains and LGG in *C. elegans* by measuring the lifespan and immune gene expression levels of worms fed individual strains.

The mean lifespans of *C. elegans* fed *E. coli* OP50 (the standard food for the worms in the lab), *S. parasanguinis* F278, *S. salivarius* F286 and LGG were 15.89 ± 0.47, 18.16 ± 0.48, 19.53 ± 0.55, and 20.3 ± 0.5 days, respectively (Figures 6A,B). Compared to *E. coli* OP50, *S. parasanguinis* F278, *S. salivarius* F286 and LGG significantly extended the average longevity of the worms by 14.3%, 23% and 27.8%, respectively (Figures 6A,B). There was no significant difference in the lifespan between *C. elegans* fed LGG and *S. salivarius* F286, indicating that *S. salivarius* F286 had a similar antiaging capacity to LGG (Figures 6A,B). The worms fed *S. salivarius* F286 and LGG lived significantly longer than those fed *S. parasanguinis* F278, suggesting that *S. salivarius* F286 and LGG had stronger lifespan-extending effects than that of *S. parasanguinis* F278 (Figures 6A,B).

The body size was measured on Day 11 after *C. elegans* began to consume the tested bacteria. Compared to *E. coli* OP50, *S. parasanguinis* F278, *S. salivarius* F286 and LGG all significantly reduced the body size of the worms (Figures 6C,D). Worms fed *S. salivarius* F286 were significantly larger in size than those fed *S. parasanguinis* F278 and LGG (Figures 6C,D).

The expression levels of immunity-related genes of *C. elegans*, which were selected based on previous studies (Supplementary Table S2), were measured by qPCR to evaluate the immune response of the worms to the two *Streptococcus* strains and LGG (Figure 6E). Compared with *E. coli* OP50, the two milk *Streptococcus* strains and LGG all upregulated the expression of antimicrobial peptide genes *cpr-1, cpr-5*, and *clec-60*. *S. salivarius* F286 and LGG also significantly increased the expression of *clec-85, lys-5* and *C15C8.3*, and *S. salivarius* F286 also tended to increase *lys-7* expression (*p* = 0.07). Compared to *S. parasanguinis* F278, *S. salivarius* F286 and LGG upregulated more antimicrobial genes, and increased the expression of *cpr-1* and *clec-60* to higher levels, suggesting that *S. salivarius* F286 and LGG exerted stronger immunostimulatory effects in *C. elegans* than *S. parasanguinis* F278. Other immune genes (*lys-8, spp-1, abf-3, nlp-29, dbl-1, pmk-1, sek-1, daf-2*) were not changed by the three bacterial strains (Figure 6E).

Quantitative PCR was performed for the antioxidative genes (Figure 6F). The two milk *Streptococcus* strains and LGG all downregulated *sod-3*. *S. salivarius* F286 and LGG significantly decreased the expression levels of *sod-2, hsp-70, trx-1, ctl-1*, and *gst-7*, but *S. parasanguinis* F278 did not. *S. salivarius* F286 also inhibited the transcription of *skn-1*. The expression of *gst-10* was significantly enhanced by all three bacterial strains to a similar extent. Only *S. salivarius* F286 upregulated *gsc-1*.

## Discussion

In the present study, the results in *in vivo* neonatal mice and *in vitro* human PBMCs and HT29 cells suggest that human breast milk *S. salivarius* F286 and *S. parasanguinis* F278 can exert a regulatory (anti-inflammatory) role in immunity, and the experiments with *C. elegans* indicate that the interaction of *S. salivarius* F286 and *S. parasanguinis* F278 with immunity is highly correlated with their lifespan-extending effect and thus may be beneficial for the host. In addition, *S. salivarius* F286 can modulate the gut microbiota composition of weaning mice. Although this study has the limitation that the *in vivo* effects of human milk *Streptococcus* strains on intestinal immunity and microbiota were evaluated in mice gut that is a different ecosystem of human gut, our work with multiple animal and human cell models highlights the potential salutary functions of human milk *Streptococcus* strains in modulating immunity and gut microbiota.

In neonatal mice, *S. salivarius* F286 and *S. parasanguinis* F278 regulated the development of gut immunity, i.e., reducing the marker gene expression of intestinal Th cells at the suckling period (age of 2 weeks) and promoting the expression of Treg cell genes at the weaning period (age of 3 weeks). It was recently found that the signature genes of CD4+ Th cells, including Th1, Th2, Th17 and Treg cells, in the homeostatic neonate intestine remained suppressed during the suckling period (first 2 weeks after birth for mice), although the neonatal intestine was rapidly colonized by a dense and dynamic microbiota after birth (44), and secretory IgA (SIgA), IgG, and epidermal growth factor (EGF) in maternal breast milk were identified to actively suppress the maturation and response of neonatal intestinal Th cells partially by reducing the translocation of bacterial antigens in the gut lumen and thus preventing neonatal immunity from being stimulated by bacterial antigens (44, 45). Here, in accordance with the dampening effect of milk antibodies and EGF on neonatal gut immunity, *S. salivarius* F286 and *S. parasanguinis* F278 reduced the ileal mRNA expression levels of cytokines and transcription factors of Th1 (IFN-γ), Th2 (Gata3 and IL-4) and Treg (TGF-β) cells in the mice at the age of week 2 (suckling period). Researchers have proposed that the synergistic suppressive effects of breast milk components on neonatal intestinal Th cells during the suckling period may promote self-tolerance, prevent autoimmunity in early life and establish immune homeostasis in adulthood (44, 46). Al Nabhani et al. recently revealed that the programmed induction of intestinal Treg cells right at weaning but not before or after weaning by the intestinal microbiota, especially Gram^+^ bacteria, guarantees the normal development of the immune system and protects the hosts from inflammatory pathologies such as colitis, allergic inflammation, and cancer later in life (47). In the current study, *S. salivarius* F286 and *S. parasanguinis* F278 upregulated the ileal expression of Treg cell-associated cytokine and transcription factor in mice at the age of week 3 (the weaning period), which indicates that the two milk *Streptococcus* strains may have the potential to contribute to the induction of gut Treg cells at the critical weaning time window.

We observed that Treg and Th1 cells responded to *S. salivarius* F286 and *S. parasanguinis* F278 in opposite ways during the suckling and weaning periods. Treg cell gene expression was suppressed at the age of 2 weeks (suckling period) but promoted at the age of 3 weeks (weaning period) by the two milk *Streptococcus* strains, and the expression of Th1 cell-related IFN-γ was downregulated at week 2 but upregulated at week 3 by *S. salivarius* F286. According to the study of Al Nabhani et al., the start of the intestinal Th1 and Treg cell programed inductions by gut bacteria right at weaning is due to the reduction of epidermal growth factor (EGF) levels in maternal breast milk (47). Researchers have found that extracellular vesicles, which are present in breast milk and serum and contain immune factors, change the proinflammatory cytokine release of dendritic cells (DCs) in response to *B. breve* and *L. rhamnosus* strains by altering Toll-like receptor 2 (TLR2) activity and phagocytosis (48). In future studies, it would be interesting to investigate whether immune components of maternal milk influence the responses of neonatal gut Th1 and Treg cells to the two milk *Streptococcus* strains.

Our results showed that *S*. s*alivarius* F286 modulated the gut microbiota at weaning (the age of 3 weeks). S. s*alivarius* F286 significantly promoted colonic bacteria from *Akkermansia* and *Intestinimonas*. *Akkermansia* strains were reported to have anti-inflammatory capabilities, fortify gut barrier function, and protect hosts from obesity, insulin resistance and colitis (23, 49). *Intestinimonas* spp. can degrade and convert Amadori reaction products (e.g., Nε-fructosyllysine), which are present in formulas due to heat treatments during milk processing and are associated with chronic diseases such as atherosclerosis, arthritis and diabetes (50), into butyrate, thus protecting formula-fed infants from undesired side effects of Amadori reaction products (51). *S. salivarius* F286 significantly inhibited bacteria from *Turicibacter*, which are putative pro-inflammatory bacteria (52). Interestingly, like LGG, *S. salivarius* F286 reduced the α-diversity of the gut microbiota of the weaning mice and decreased bacteria from *Eubacterium, Bifidobacterium, Ruminiclostridium, Escherichia*-*Shigella*, and Lachnospiraceae, indicating that *S. salivarius* F286 and LGG may have similar mechanism(s) to inhibit these gut bacteria. Indeed, while LGG secretes antibacterial peptides with anti-gram-negative and anti–gram-positive bactericidal activity (53), *S. salivarius* produces broad-spectrum bacteriocins (54); they both produce lactic acids, and *Lactobacillus* have been shown to outcompete bacteria, including *Bifidobacterium*, at low pH levels (55). The probiotic *S. salivarius* K12 strain was also reported to decrease the α-diversity of the oral microbiota of periodontitis mice (56).

*S. parasanguinis* F278 did not change the colonic microbiota composition of weaning pups, probably because *S. parasanguinis* F278 replaced the endogenous *S. parasanguinis* strain, as shown by our qPCR and 16S rRNA gene sequencing results, and the two *S. parasanguinis* strains may have very similar, if not identical, ecological niches in the ecosystem of the pups' gut microbiota. At the age of 2 weeks, *S. salivarius* F286, *S. parasanguinis* F278 and LGG did not change the pups' gut microbiota, probably because dam breast milk exerts a predominant shaping effect on the gut microbiota at this suckling period. Indeed, researchers have found that the cessation of breastfeeding rather than the introduction of solid foods makes the infant gut microbiota develop into an adult microbiota during the weaning process (57).

*S. salivarius* F286 and *S. parasanguinis* F278 promoted the expression of ileal Treg cell genes in weaning mice, induced anti-inflammatory cytokine secretion profiles in human adult PBMCs, and reduced TNF-α-induced inflammation of human gut epithelial HT29 cells. These results indicate the potential of *S. salivarius* F286 and *S. parasanguinis* F278 to alleviate inflammatory or allergic diseases, because Treg cells are protective against atopic (58) and inflammatory diseases (47), and some *Bifidobacterium* and *Lactobacillus* strains that are shown to have anti-inflammatory and Treg cell-promoting capacities protect mice/rats against colitis (25, 59) and allergies (60, 61). Viridans streptococci, which include *S. salivarius*, were found to be less prevalent in the gut of infants with atopic eczema than in healthy infants (62). In previous reports, the live cells or culture supernatants of *S. salivarius* strains isolated from the human oral cavity, feces, pharynges and blood reduced the NF-κB activation and pro-inflammatory IL-8 production of human gingival, bronchial, pharyngeal, and intestinal epithelial cells *in vitro* stimulated with pro-inflammatory cytokines or pathogens (24, 43, 63, 64), and one oral *S. salivarius* strain significantly alleviated trinitrobenzenesulfonic acid (TNBS)-induced colitis in mice (24). The results of previous and our studies warrant further investigations on the effects of milk *Streptococcus* strains, especially *S. salivarius* strains, on inflammatory and allergic diseases. Lactate, the main product of *Streptococcus* species in carbohydrate fermentation (65), has been shown to inhibit inflammation by binding to the receptor GPR81 expressed on the surface of innate immune cells (66) and gut epithelial cells (67). Therefore, the anti-inflammatory properties of *S. salivarius* F286 and *S. parasanguinis* F278 may be attributed to their lactate production, but this needs to be proved in further study by comparing the anti-inflammatory capacity between bacterial culture supernatants and blank bacterial culture medium supplemented with lactate of similar concentrations of culture supernatants, and between wild type *Streptococcus* strains and strains deficient in lactate production [e.g., strains lacking lactate dehydrogenase activity (68)].

*S. salivarius* F286 stimulated both proinflammatory Th1 cells and anti-inflammatory Treg cells in the ileum of weaning mice at the age of week 3, and its heat-killed cells induced human PBMCs to concurrently produce the Th1 cytokine IL-12 and the anti-inflammatory cytokine IL-10, indicating that *S. salivarius* F286 may be able to play both immunostimulatory and anti-inflammatory roles in immunity. This is consistent with the reports of van den Bogert et al. who found that four *S. salivarius* strains isolated from human small intestine induced human monocyte-derived immature dendritic cells (iDCs) to secrete both the Th1 cytokine IL-12 and the anti-inflammatory cytokine IL-10 in *in vitro* experiments (69). Human breast milk-derived *L. fermentum* CECT5716 was also shown to stimulate anti-inflammatory Treg cell differentiation and IL-10 production and to activate proinflammatory Th1 cytokine release (25, 70); correspondingly, ingestion of *L. fermentum* CECT5716 not only prevented colitis and septic shock in rodents (71, 72) but also promoted the immune response of human subjects to influenza vaccination by increasing the Th1 response (73).

At the age of 2 weeks, *S. salivarius* F286 and *S. parasanguinis* F278 showed suppressive effects on ileal Th cells mainly in males and females, respectively, indicating that male and female pups responded differently to the two milk *Streptococcus* strains. This is in accordance with previous reports that different sexes have differences in immune responses that exist from birth (74) and that some immune genes (e.g., *Nos2, Mcpt1, Mcpt2, Ccr3*) in the small intestine of 2-week-old mice show sexually dimorphic expression (75). This warrants further studies to investigate whether the mixture of *S. salivarius* F286 and *S. parasanguinis* F278 can suppress Th cells of suckling mouse pups of both genders.

In *C. elegans* experiments, the lifespan-extending effects of *S. salivarius* F286, *S. parasanguinis* F278 and LGG were closely associated with their activation of antimicrobial peptide genes. The two milk *Streptococcus* strains and LGG all upregulated the expression of *cpr-*1 and *cpr-5, S. salivarius* F286 tended to increase *lys-7* expression, and the activation of these antimicrobial peptide genes has been reported to contribute to the lifespan of *C. elegans* being extended (28, 76, 77). Note also that compared to *S. parasanguinis* F278, *S. salivarius* F286 and LGG, which increased the expression of *cpr-1* to higher levels and upregulated more antimicrobial peptide genes, prolonged the lifespan longer. Together, these observations indicate that the interactions of the two milk *Streptococcus* strains and LGG with immunity may contribute to their probiotic effects in worms.

Because dietary/calorie restriction significantly reduces the body size of the worms when prolonging the lifespan (78), and *S. salivarius* F286, *S. parasanguinis* F278 and LGG decreased the body size of the worms, dietary restriction contributed to the prolonging effect of the two *Streptococcus* strains in *C. elegans*. However, the expression levels of antioxidant genes (*sod-2, hsp-70, trx-1, skn-1*), which were reported to be significantly upregulated in worms under dietary/calorie restriction induced by genetic mutation (79) or food restriction (78, 80, 81), were significantly downregulated in *S. salivarius* F286- and LGG-fed worms and were not changed in *S. parasanguinis* F278-fed worms. In addition, the expression levels of *clec-85* and genes of the p38 signaling pathway, such as *pmk-1* and *sek-1*, which were found to be downregulated by dietary restriction (82), were enhanced or remained unchanged in *S. salivarius* F286-, *S. parasanguinis* F278-, and LGG-fed worms. These results suggest that dietary/calorie restriction was not the only mechanism through which the two *Streptococcus* strains and LGG prolonged the lifespan of *C. elegans*.

In conclusion, human breast milk *S. salivarius* F286 and *S. parasanguinis* F278 may exert regulatory (anti-inflammatory) roles in gut immunity, and *S. salivarius* F286 can modulate gut microbiota at weaning. Our results suggest that the two milk *Streptococcus* strains have probiotic potential and might be able to enhance host immune tolerance at early life and resistance to inflammatory pathologies in adulthood, and therefore warrant further study on the preventing/treating effects of milk *S. salivarius* and *S. parasanguinis* strains on inflammatory pathologies such as colitis and allergic inflammation.

## Data Availability Statement

The datasets presented in this study can be found in online repositories. The names of the repository/repositories and accession number(s) can be found at: https://www.ncbi.nlm.nih.gov/sra/?term=srp306510, SRP306510, https://www.ncbi.nlm.nih.gov/nuccore/KY038192.1/, https://www.ncbi.nlm.nih.gov/nuccore/KY038191.1/, https://www.ncbi.nlm.nih.gov/nuccore/AP011548.1/.

## Ethics Statement

The protocols and procedures of human PBMC collection and processing were approved by the Ethical Committee of Putuo People's Hospital of Zhou Shan City, Zhejiang province, China (No. 2020KY05), and written informed consent was obtained from the volunteer before the participation in the study. The animal study was reviewed and approved by Institutional Animal Care and Use Committee (IACUC), School of Life Sciences and Biotechnology, Shanghai Jiao Tong University.

## Author Contributions

JS, SL, and NL designed the study. SL, CW, ZZ, YL, XY, CC, LW, and JS performed the mouse experiment and sample collection. SL, XL, and YiZ performed the DNA extraction, 16S rRNA gene sequencing, sequencing data analysis, qPCR with the colon content DNA and mouse ileal tissues, and analysis on villus length and body weight. NL, JC, XW, and YuZ performed *C. elegans* experiment. NL extracted RNA from the worms, performed qPCR and analyzed the data from the *C. elegans* experiment, and measured cytokines and performed the data analysis. NL and SL conducted the PBMC and HT29 cell assays. JS, NL, SL, and LZ wrote and revised the manuscript. All authors contributed to the article and approved the submitted version.

## Funding

This work was supported by the National Natural Science Foundation of China (81570809 and 81661128010).

## Conflict of Interest

The authors declare that the research was conducted in the absence of any commercial or financial relationships that could be construed as a potential conflict of interest.

## Publisher's Note

All claims expressed in this article are solely those of the authors and do not necessarily represent those of their affiliated organizations, or those of the publisher, the editors and the reviewers. Any product that may be evaluated in this article, or claim that may be made by its manufacturer, is not guaranteed or endorsed by the publisher.
