# Supplementary material for: Gut Microbiota and Immune Modulatory Properties of Human Breast Milk Streptococcus salivarius and S. parasanguinis Strains
Source: Front Nutr. 2022 Feb 22;9:798403. doi: 10.3389/fnut.2022.798403 (PMC8901577; doi:10.3389/fnut.2022.798403)
Supplement: Supplementary file 1 [file Data_Sheet_1.docx]

**Gut Microbiota and Immune Modulatory Properties of Human Breast Milk *Streptococcus salivarius* and *S. parasanguinis* Strains**

**Supplementary Tables**

**Supplementary Table S1 Primer pairs used to measure ileal immune gene expression levels and amounts of colonic *S. salivarius*, *S. parasanguinis* and LGG in mice with quantitative real-time PCR**

|  | Gene | Sequences (5’-3’) | Annealing  temperature (°C) | Reference |  |
| --- | --- | --- | --- | --- | --- |
| Mouse ileum genes | | | | |  |
| T cell-related genes | *T-bet* | F: AGCAAGGACGGCGAATGTT  R: GGGTGGACATATAAGCGGTTC | 56 | [1] |  |
|  | *Ifn-γ* | F: AAGCGTCATTGAATCACACCTG  R: TGACCTCAACTTGGCAATACTC | 56 | [1] |  |
|  | *Gata3* | F: CTCGGCCATTCGTACATGGAA  R: GGATACCTCTGCACCGTAGC | 56 | [1] |  |
|  | *Il-4* | F: GGTCTCAACCCCCAGCTAGT  R: GCCGATGATCTCTCTCAAGTGAT | 56 | [1] |  |
|  | *Foxp3* | F: ACCATTGGTTTACTCGCATGT  R: TCCACTCGCACAAAGCACTT | 56 | [2] |  |
|  | *Tgfβ* | F: AAGTTGGCATGGTAGCCCTT  R: GCCCTGGATACCAACTATTGC | 56 | [2] |  |
|  | *Rorγt* | F: ACGGCCCTTGGTTCTCATCA  R: CCAAATTGTATTGCAGATGTTCCAC | 56 | [1] |  |
|  | *Il-10* | F: GGGGCCAGTACAGCCGGGAA  R: CTGGCTGAAGGCAGTCCGCA | 56 | [1] | |
| Anti-microbial peptide genes | *Defβ1* | F: AGGTGTTGGCATTCTCACAAG  R: GCTTATCTGGTTTACAGGTTCCC | 56 | NM_007843.3 |  |
|  | *RegⅢγ* | F: ATGCTTCCCCGTATAACCATCA  R: GGCCATATCTGCATCATACCAG | 56 | NM_011260.2 |  |
| House-keeping gene | *β-actin* | F: GGC TGTATTCCCCTCCATCG  R: CCAGTTGGTAACAATGCCATGT | 56 | [2] |  |
| Bacterial target genes | | | | |  |
| Total bacterial quantity primers | 16S rRNA gene | F: TCCTACGGGAGGCAGCAGT  R: GGACTACCAGGGTATCTAATCCTGTT | 56 | [2] |  |
| *S. salivarius*-specific primers | *MAP* | F: GGGAAGCATTATGGATTACC  R: ACACATCAAGGACTGACTTATC | 65 | [3] |  |
| *S. parasanguinis*-specific primers | *groEL* | F: AACAATGCGATYCCAGTATCRAG*  R: CTACGACATTAAAGGTACCDCGG* | 59 | [4] |  |
| LGG-specific primers | Phage-related gene | F: GTCAACCATTGCTTGCCCTT  R: AGAAAAGACACCGGGTTTGC | 65 | [5] |  |
|  |  |  |  |  |  |

^*^, Y(C or T), R(A or G), D(G or A or T).,

**Supplementary Table S2 Primer pairs used to measure gene expression levels of *C. elegans* with quantitative real-time PCR**

| **Gene** | **Primer** | **Sequence** | **Annealing**  **temperature (°C)** | **Reference** |
| --- | --- | --- | --- | --- |
| *cpr-1* | forward | 5'-AAAGTACCTCGGAGGACACG-3' | 56 | [6] |
|  | reverse | 5'-ATTCCGCATTGATCATCTCC-3' |  |  |
| *cpr-5* | forward | 5'-GACAACGGAACCCCATACTG-3' | 56 | [6] |
|  | reverse | 5'-CTCGATTCCACACTCGTTGA-3' |  |  |
| *clec-60* | forward | 5'-ACGGGCAAGTTATTGGAGAG-3' | 56 | [6] |
|  | reverse | 5'-ACACGGTATTGAATCCACGA-3' |  |  |
| *clec-85* | forward | 5'-CCTGATGATAAGTATATTGGAGACCTGTGCTACTC-3' | 56 | [7] |
|  | reverse | 5'-GGTTTTGGCTGTAGCACGCCGACTGAGCATCC-3' |  |  |
| *lys-5* | forward | 5'-TCCCAGAATTTATCATTCATCG-3' | 56 | [6] |
|  | reverse | 5'-TGGCATTCTTGACATTTTGC-3' |  |  |
| *lys-7* | forward | 5'-ATTCAGGTCACTTCGCCAAC-3' | 56 | [8] |
|  | reverse | 5'-ATCCGGTCGTGATCTGATTC-3' |  |  |
| *lys-8* | forward | 5'-CTCCACGAGTTCCACCAAAT-3' | 56 | [8] |
|  | reverse | 5'-TATGCACGGACAAAGACTGC-3' |  |  |
| *spp-1* | forward | 5'-GGCTCTCGTCGAGGGTGGAGAG-3' | 56 | [7] |
|  | reverse | 5'-CACACTCGTGATGCAACGGCAACAGC-3' |  |  |
| *abf-3* | forward | 5'-GGTGTCGAATAAGGCAGTGTGGACCT-3' | 56 | [7] |
|  | reverse | 5'-GGCATTTCCATAGCTATCCCTGTAGC-3' |  |  |
| *nlp-29* | forward | 5'-GGGGATATGGAGGATATGGAAGAGGATATGG-3' | 56 | [7] |
|  | reverse | 5'-CCGTATCCTCCGTACATTCCACGT-3' |  |  |
| *dbl-1* | forward | 5'-GCCATTCTCCACCTCTTCCT-3' | 56 | [8] |
|  | reverse | 5'-GGAACATCAATGCTCGGACC-3' |  |  |
| *pmk-1* | forward | 5'-ACTCGCCGTGATTTCAAACG-3' | 56 | [8] |
|  | reverse | 5'-CAGTTGGACGACGATCTGGA-3' |  |  |
| *sek-1* | forward | 5'-TGGCAAACACATTCCAGAGC-3' | 56 | [8] |
|  | reverse | 5'-AGTCTTGGCCATGCTGTTTG-3' |  |  |
| *daf-2* | forward | 5'-GCCCGAATGTTGTGAAAACT-3' | 56 | [8] |
|  | reverse | 5'-CCAGTGCTTCTGAATCGTCA-3' |  |  |
| *C15C8.3* | forward | 5’-ATCGGAGCACCAAAGAGTGT-3’ | 56 | [6] |
|  | reverse | 5’-AAGAATGCACCCGTAAGTGG-3’ |  |  |
| *sod-1* | forward | 5'-CGTAGGCGATCTAGGAAATGTG-3' | 56 | [9] |
|  | reverse | 5'-AACAACCATAGATCGGCCAACG-3' |  |  |
| *sod-2* | forward | 5'-CTTCAAAACACCGTTCGCTG-3' | 56 | [9] |
|  | reverse | 5'-CAGTGGAACAAGTCCAGTT-3' |  |  |
| *sod-3* | forward | 5'-TTCAAAGGAGCTGATGGACACT-3' | 56 | [9] |
|  | reverse | 5'-AAGTGGGACCATTCCTTCCAA-3' |  |  |
| *hsp-70* | forward | 5'-ACGGGATGCTGTCATTACTG-3' | 56 | [9] |
|  | reverse | 5'-ATGTACCTCCTCCCAAATCG-3' |  |  |
| *trx-1* | forward | 5'-TCCAACACTTTTTGACGCAG-3' | 56 | [9] |
|  | reverse | 5'-CAAGATGATGCCGACTTTCA-3' |  |  |
| *ctl-1* | forward | 5'-GCGGATACCGTACTCGTGAT-3' | 56 | [9] |
|  | reverse | 5'-GTGGCTGCTCGTAGTTGTGA-3' |  |  |
| *skn-1* | forward | 5'-CTCTCTTCTGGCATCCTCTACCA-3' | 56 | [9] |
|  | reverse | 5'-TTCTTGGATTCTTCTTCTTGTTCGT-3' |  |  |
| *gst-4* | forward | 5'-GATGCTCGTGCTCTTGCTG-3' | 56 | [9] |
|  | reverse | 5'-CCGAATTGTTCTCCATCGAC-3' |  |  |
| *gst-7* | forward | 5'-GGACAAGACTTCGAGGACAAC-3' | 56 | [9] |
|  | reverse | 5'-AACTGACGAGCCAAGTAACG-3' |  |  |
| *gst-10* | forward | 5'-AAGAGATTGTGCAGACTGGAG-3' | 56 | [9] |
|  | reverse | 5'-AGAACATGTCGAGGAAGGTTG-3' |  |  |
| *gcs-1* | forward | 5'-ATTTCTGGAGCATCTGGTGG-3' | 56 | [9] |
|  | reverse | 5'-AAGGTAGTCCGTTGACGTGG-3' |  |  |
| *hsp-16.2* | forward | 5'-CTATTTCCGTCCAGCTCAAC-3' | 56 | [9] |
|  | reverse | 5'-TTTGTTCAACGGGCGCTTGC-3' |  |  |
| *clk-1* | forward | 5'-GCAATAGCTCCCTTGCATCC-3' | 56 | [9] |
|  | reverse | 5'-AGCACATACTGCTGCTTCTC-3' |  |  |
| *act-1* | forward | 5'-GCTGGACGTGATCTTACTGATTACC-3' | 56 | [9] |
|  | reverse | 5'-GTAGCAGAGCTTCTCCTTGATGTC-3' |  |  |

**Supplementary Table S3 Numbers and prevalence of sequences of ASVs identified as *S. salivarius*, *S. parasanguinis* and LGG in the colon contents of each animal group^#^**

| Bacteria | ASV | 2-week-old pups | | | | | | | | 3-week-old pups | | | | | | | | |
| --- | --- | --- | --- | --- | --- | --- | --- | --- | --- | --- | --- | --- | --- | --- | --- | --- | --- | --- |
|  |  | CTL | | SsaF286 | | SpaF278 | | LGG | | CTL | | SsaF286 | | | SpaF278 | | LGG | |
|  |  | Seq  No. ^a^ | Prev^b^ | Seq  No.^a^ | Prev^b^ | Seq  No.^a^ | Prev ^b^ | Seq  No.^a^ | Prev^b^ | Seq  No.^a^ | Prev ^b^ | | Seq  No.^a^ | Prev^b^ | Seq  No.^a^ | Prev ^b^ | Seq No.^a^ | Prev ^b^ |
| *S. salivarius* | ASV97 (100% identical with *S. salivarius* F286) | 0  (0-0) | 0/15 | 112.5  (46-372) | 16/16 | 0  (0-0) | 0/18 | 0  (0-2) | 1/14 | 0  (0-0) | 0/17 | | 5  (0-12) | 13/19 | 0  (0-0) | 0/19 | 0  (0-0) | 0/13 |
| *S. parasanguinis* | ASV202 (100% identical with *S. parasanguinis* F278) | 0  (0-0) | 0/15 | 0  (0-0) | 0/16 | 28.5  (5-268) | 18/18 | 0  (0-0) | 0/14 | 0  (0-0) | 0/17 | | 0  (0-0) | 0/19 | 0  (0-13) | 2/19 | 0  (0-0) | 0/13 |
|  | ASV31 (100% identical with *S. parasanguinis* KCOM strain, but 97.9% similar with *S. parasanguinis* F278) | 253  (0-732) | 9/15 | 0  (0-0) | 0/16 | 0  (0-301) | 4/18 | 23.5  (0-2561) | 7/14 | 0  (0-4) | 2/17 | | 0  (0-0) | 0/19 | 0  (0-0) | 0/19 | 0  (0-35) | 3/13 |
| *L. rhamnosus* | ASV49 (100% identical with LGG) | 0  (0-2) | 1/15 | 0  (0-0) | 0/16 | 0  (0-0) | 0/18 | 297  (31-1278) | 14/14 | 0  (0-0) | 0/17 | | 0  (0-0) | 0/19 | 0  (0-0) | 0/19 | 0  (0-25) | 4/13 |

^a^, Seq No. represents the number of sequences of the ASV in the total 24000 (downsized) sequences of colon contents samples of individual animal groups. The number of sequences of each ASV is shown as the median number (minimum - maximum) of each animal group.

^b^, Prev represents the prevalence of the ASV in each animal group, which is the number of animals having the ASV relative to total animal number of the group.

**^#^**, CTL, Control group; SsaF286, *S. salivarius* F286 group; SpaF278, *S. parasanguinis* F278 group; LGG, LGG group.

**Supplementary Table S4 The comparison of the numbers of 16S rRNA gene sequences of two *S. parasanguinis* ASVs (ASV31 and ASV202) determined with Illumina sequencing and *S. parasanguinis* quantities determined as copies of *groEL* gene/g colon content with qPCR in individual mice at the age of 2 weeks**

| **Litter**  **ID** | **Mouse ID** | **Group** | **Sequence number of**  **ASV31**  **(97.9% similar with *S. parasanguinis* F278) *** | **Sequence**  **number of**  **ASV202**  **(100% identical with**  ***S. parasanguinis* F278) ^§^** | **Sum of sequence numbers of ASV31 and ASV202 ^#^** | ***S. paransanguinis* amount determined with qPCR (copies of *groEL* gene/g colon content)** | **ASV31:ASV202**  **Ratio ^¶^** |
| --- | --- | --- | --- | --- | --- | --- | --- |
| B4 | B401 | SpaF278 | 26 | 5 | 31 | 3.39E+09 | 5.2 |
|  | B403 | SpaF278 | 131 | 28 | 159 | 3.78E+09 | 4.7 |
| B5 | B502 | SpaF278 | 230 | 12 | 242 | 3.62E+09 | 19.2 |
|  | B503 | SpaF278 | 301 | 21 | 322 | 6.44E+09 | 14.3 |
| B1 | B102 | SpaF278 | 0 | 75 | 75 | 7.90E+09 | 0 |
|  | B107 | SpaF278 | 0 | 43 | 43 | 4.31E+09 | 0 |
| B2 | B203 | SpaF278 | 0 | 268 | 268 | 3.87E+09 | 0 |
|  | B207 | SpaF278 | 0 | 8 | 8 | 5.91E+08 | 0 |
| B3 | B301 | SpaF278 | 0 | 37 | 37 | 1.61E+09 | 0 |
|  | B302 | SpaF278 | 0 | 19 | 19 | 1.14E+09 | 0 |
| B6 | B601 | SpaF278 | 0 | 48 | 48 | 2.10E+09 | 0 |
|  | B606 | SpaF278 | 0 | 15 | 15 | 1.46E+09 | 0 |
| B7 | B701 | SpaF278 | 0 | 39 | 39 | 1.50E+09 | 0 |
|  | B707 | SpaF278 | 0 | 50 | 50 | 5.36E+09 | 0 |
| B8 | B801 | SpaF278 | 0 | 35 | 35 | 3.23E+09 | 0 |
|  | B803 | SpaF278 | 0 | 21 | 21 | 6.56E+09 | 0 |
| B9 | B903 | SpaF278 | 0 | 14 | 14 | 2.59E+09 | 0 |
|  | B905 | SpaF278 | 0 | 29 | 29 | 2.82E+09 | 0 |
| C3 | C301 | CTL | 267 | 0 | 267 | 7.68E+09 | Undefined |
|  | C305 | CTL | 418 | 0 | 418 | 9.65E+09 | Undefined |
| C4 | C401 | CTL | 272 | 0 | 272 | 1.19E+10 | Undefined |
|  | C402 | CTL | 253 | 0 | 253 | 1.33E+10 | Undefined |
| C6 | C601 | CTL | 303 | 0 | 303 | 2.06E+10 | Undefined |
|  | C603 | CTL | 732 | 0 | 732 | 1.26E+10 | Undefined |
| C7 | C701 | CTL | 475 | 0 | 475 | 6.07E+09 | Undefined |
|  | C702 | CTL | 215 | 0 | 215 | 8.18E+09 | Undefined |
|  | C704 | CTL | 305 | 0 | 305 | 5.66E+09 | Undefined |
| C1 | C102 | CTL | 0 | 0 | 0 | 4.09E+05 | Not Applicable |
|  | C104 | CTL | 0 | 0 | 0 | 6.82E+05 | Not Applicable |
| C2 | C201 | CTL | 0 | 0 | 0 | 0.00E+00 | Not Applicable |
|  | C209 | CTL | 0 | 0 | 0 | 0.00E+00 | Not Applicable |
| C5 | C501 | CTL | 0 | 0 | 0 | 0.00E+00 | Not Applicable |
|  | C503 | CTL | 0 | 0 | 0 | 1.43E+06 | Not Applicable |
| D5 | D501 | LGG | 248 | 0 | 248 | 5.91E+09 | Undefined |
|  | D502 | LGG | 523 | 0 | 523 | 5.65E+09 | Undefined |
| D6 | D602 | LGG | 1355 | 0 | 1355 | 1.48E+10 | Undefined |
|  | D603 | LGG | 2561 | 0 | 2561 | 1.92E+10 | Undefined |
| D7 | D701 | LGG | 50 | 0 | 50 | 6.04E+09 | Undefined |
|  | D703 | LGG | 47 | 0 | 47 | 4.57E+09 | Undefined |
| D8 | D804 | LGG | 754 | 0 | 754 | 1.21E+09 | Undefined |
| D1 | D106 | LGG | 0 | 0 | 0 | 3.71E+06 | Not Applicable |
| D2 | D202 | LGG | 0 | 0 | 0 | 2.85E+07 | Not Applicable |
|  | D205 | LGG | 0 | 0 | 0 | 3.79E+06 | Not Applicable |
| D3 | D301 | LGG | 0 | 0 | 0 | 4.52E+06 | Not Applicable |
|  | D304 | LGG | 0 | 0 | 0 | 7.40E+06 | Not Applicable |
| D4 | D401 | LGG | 0 | 0 | 0 | 3.51E+06 | Not Applicable |
|  | D402 | LGG | 0 | 0 | 0 | 2.64E+06 | Not Applicable |
| A1 | A106 | SsaF286 | 0 | 0 | 0 | 3.26E+06 | Not Applicable |
| A2 | A204 | SsaF286 | 0 | 0 | 0 | 0.00E+00 | Not Applicable |
|  | A205 | SsaF286 | 0 | 0 | 0 | 3.75E+06 | Not Applicable |
| A3 | A301 | SsaF286 | 0 | 0 | 0 | 2.27E+06 | Not Applicable |
|  | A302 | SsaF286 | 0 | 0 | 0 | 2.81E+06 | Not Applicable |
| A4 | A403 | SsaF286 | 0 | 0 | 0 | 9.58E+04 | Not Applicable |
|  | A405 | SsaF286 | 0 | 0 | 0 | 6.19E+05 | Not Applicable |
| A5 | A501 | SsaF286 | 0 | 0 | 0 | 1.11E+06 | Not Applicable |
|  | A502 | SsaF286 | 0 | 0 | 0 | 0.00E+00 | Not Applicable |
| A6 | A601 | SsaF286 | 0 | 0 | 0 | 1.98E+06 | Not Applicable |
|  | A604 | SsaF286 | 0 | 0 | 0 | 1.65E+06 | Not Applicable |
| A7 | A702 | SsaF286 | 0 | 0 | 0 | 0.00E+00 | Not Applicable |
|  | A704 | SsaF286 | 0 | 0 | 0 | 1.69E+06 | Not Applicable |
| A8 | A802 | SsaF286 | 0 | 0 | 0 | 5.68E+04 | Not Applicable |
|  | A806 | SsaF286 | 0 | 0 | 0 | 3.71E+05 | Not Applicable |
| A9 | A902 | SsaF286 | 0 | 0 | 0 | 8.75E+04 | Not Applicable |

^#^, The sum of sequence numbers of two *S. parasanguinis* ASVs (ASV31 and ASV202) was significantly correlated with the amounts of *S. paransanguinis* determined with qPCR (Supplementary Figure S3a, b)

*, In 16 mice in which only ASV31 was detected (highlighted by blue), ASV31 sequence numbers were significantly correlated with the *S. paransanguinis* amounts determined with qPCR (Supplementary Figure S3c).

**^§^**, In 14 mice in which only ASV202 was detected (highlighted by yellow), the sequence numbers of ASV202 were significantly correlated with the amounts of *S. paransanguinis* determined with qPCR (Supplementary Figure S3d).

**^¶^**, In 30 out of 34 mice in which either ASV31 or ASV202 was detected (highlighted by blue and yellow), the two ASVs did not appear simultaneously; in the only 4 mice in which both ASVs were present (highlighted by green), the ratio of the two ASVs varied by about 5 folds.

**Supplementary Table S5 P values of PERMANOVA tests based on weighted UniFrac distance between male and female mice in each animal group at the age of 2 and 3 weeks**^a^

|  |  | **2-week-old** | **3-week-old** |
| --- | --- | --- | --- |
|  | **Group** ^b^ | **p-value** | **p-value** |
| **Male *vs*. Female** | CTL | 0.205 | 0.550 |
|  | SsaF286 | 0.47 | 0.065 |
|  | SpaF278 | 0.922 | 0.997 |
|  | LGG | 0.839 | 0.500 |

^a^, In each animal group, the structure of colonic microbiota did not show gender difference according to the PERMANOVA tests based on weighted UniFrac distance

^b^, CTL, Control group; SsaF286, *S. salivarius* F286 group; SpaF278, *S. parasanguinis* F278 group; LGG, LGG group.

References

1. Zhao L, Yang S, Guo Y, Sun G, Li B. Chronic arsenic exposure in drinking water interferes with the balances of T lymphocyte subpopulations as well as stimulates the functions of dendritic cells in vivo. Int Immunopharmacol. 2019; 71:115-131.

2. Zhai R, Xue X, Zhang L, Yang X, Zhao L, Zhang C. Strain-Specific Anti-inflammatory Properties of Two Akkermansia muciniphila Strains on Chronic Colitis in Mice. Front Cell Infect Microbiol. 2019; 9:239.

3. Faraji R, Behjati-Ardakani M, Faraji N, Moshtaghioun SM, Kalantar SM, Pedarzadeh A, Zandi H, Sarebanhassanabadi M, Ahmadi N, Dehghani Firoozabadi A. Molecular Diagnosis of Bacterial Definite Infective Endocarditis by Real-Time Polymerase Chain Reaction. Cardiol Res. 2018; 9(2):99-106.

4. Chen Q, Wu G, Chen H, Li H, Li S, Zhang C, Pang X, Wang L, Zhao L, Shen J. Quantification of Human Oral and Fecal Streptococcus parasanguinis by Use of Quantitative Real-Time PCR Targeting the groEL Gene. Front Microbiol. 2019; 10:2910.

5. Laursen MF, Laursen RP, Larnkjaer A, Michaelsen KF, Bahl MI, Licht TR. Administration of two probiotic strains during early childhood does not affect the endogenous gut microbiota composition despite probiotic proliferation. BMC Microbiol. 2017; 17(1):175.

6. Kim Y, Mylonakis E. Caenorhabditis elegans immune conditioning with the probiotic bacterium Lactobacillus acidophilus strain NCFM enhances gram-positive immune responses. Infect Immun. 2012; 80(7):2500-2508.

7. Alper S, McBride SJ, Lackford B, Freedman JH, Schwartz DA. Specificity and complexity of the Caenorhabditis elegans innate immune response. Mol Cell Biol. 2007; 27(15):5544-5553.

8. Kwon G, Lee J, Lim YH. Dairy Propionibacterium extends the mean lifespan of Caenorhabditis elegans via activation of the innate immune system. Sci Rep. 2016; 6:31713.

9. Nakagawa H, Shiozaki T, Kobatake E, Hosoya T, Moriya T, Sakai F, Taru H, Miyazaki T. Effects and mechanisms of prolongevity induced by Lactobacillus gasseri SBT2055 in Caenorhabditis elegans. Aging Cell. 2016; 15(2):227-236.
