# Supplementary material for: Gut Microbiota and Immune Modulatory Properties of Human Breast Milk Streptococcus salivarius and S. parasanguinis Strains
Source: Front Nutr. 2022 Feb 22;9:798403. doi: 10.3389/fnut.2022.798403 (PMC8901577; doi:10.3389/fnut.2022.798403)
Supplement: Supplementary file 2 [file Data_Sheet_2.docx]

Gut Microbiota and Immune Modulatory Properties of Human Breast Milk *Streptococcus salivarius* and *S. parasanguinis* Strains

**Supplementary Materials and Methods**

**Isolation of *Lactobacillus rhamnosus* GG (LGG)**

One packet of Culturelle^®^ probiotic powder during its shelf-life was suspended in sterile Mann-Rogosa Sharpe (MRS) broth medium (Hopebiol, Qingdao, China), and 500 μl bacterial suspension was inoculated into 50 ml freshly prepared MRS broth and cultured for 24 hours in an anaerobic workstation (DG500, DWS, United Kingdom) at 37 °C. The culture was then streaked on MRS agar medium and anaerobically grown at 37 °C for 24 hours. A single colony was picked and purified by plate streaking three times. The full-length 16S rRNA gene of the single colony was PCR amplified with universal primers 27f (5’-AGAGTTTGATCCTGGCTCAG) and 1492r (5’- GGTTACCTTGTTACGACTT), ligated into the pGEM-T Easy vector (Promega, US), transformed into *E. coli* DH5α competent cells, sequenced bidirectionally using T7 (5'-TAATACGACTCACTATAGGG-3') and SP6 (5'-ATTTAGGTGACACTATAG-3') sequencing primers on an ABI 3730XL sequencer (Applied Biosystems), and assembled into consensus sequences with a CodonCode Aligner (Codon Code Corporation). The cloned 16S rRNA gene sequences were blasted against the nr database of GenBank using the basic local alignment search tool (BLAST) and showed 100% identity with that of LGG (ATCC53103), confirming that the single colony we isolated from the Culturelle^®^ probiotic powder was LGG.

**The preparation of live bacterial cell suspensions for the mouse experiment**

The cells of *S. salivarius* F286, *S. parasanguinis* F278, and LGG were harvested by centrifuging their broth cultures at 9000 ×g for 5 min, washed twice with sterile phosphate-buffered saline (PBS) (Gibco, USA), and finally suspended in PBS containing 10% Difco^TM^ skim milk (BD, USA) at the concentration of 1×10^9^ CFU/ml. Aliquots of bacterial suspensions were stored at -80 °C until use.

**The preparation of the bacterial culture supernatants and heat-killed bacterial cell suspensions for *in vitro* human cell experiments**

Broth cultures of *S. parasanguinis* F278, *S. salivarius* F286 and LGG were centrifuged at 5,000 ×g for 10 min. The bacterial culture supernatants were pipetted into different tubes and filtered through 0.22 μm filters (Merck Millipore, MA, USA). The bacterial pellets were washed twice by resuspension in sterile PBS and centrifugation at 5,000 ×g for 10 min. The washed bacterial cells were resuspended in sterile PBS, diluted to multiple working concentrations of 4×10^7^, 1×10^8^, 4×10^8^, 1×10^9^, 1.6×10^9^ and 4×10^9^ CFU/ml, and heat-killed by heating at 65 °C for 20 min (1). Aliquots of heat-killed bacterial cell suspensions and filtered bacterial culture supernatants were stored at -20 °C until use.

**The preparation of live bacterial cell pellets for *C. elegans* experiments**

For *C. elegans* experiments, broth cultures of *S. parasanguinis* F278, *S. salivarius* F286, LGG and *E. coli* OP50 were centrifuged at 15,000 ×g for 10 min, and the resultant bacterial cell pellets were washed twice by resuspension in sterile M9 buffer and centrifugation at 15,000 ×g for 10 min. The washed bacterial cell pellets were weighed and resuspended in M9 buffer at a concentration of 10 mg/100 μl. Then, 100 μl freshly prepared bacterial suspensions of each bacterial strain were spread on modified nematode growth medium (mNGM) that contained no peptone in 60-mm-diameter plates to feed the worms.

**Sample collection of the mouse experiment**

At the age of 2 weeks and 3 weeks, one male and one female of individual litters were randomly picked and sacrificed. Ileum was collected and one fragment was fixed in 4% paraformaldehyde, embedded in paraffin, and subjected to hematoxylin and eosin staining, and the other fragment was snap-frozen in liquid nitrogen and stored at -80 °C for subsequent RNA extraction and gene expression quantification. Colon was collected. The colon tissue was fixed in 4% paraformaldehyde, embedded in paraffin, and subjected to hematoxylin and eosin (H&E) staining, and the colon content was snap-frozen in liquid nitrogen and stored at -80 °C until gut microbiota analysis.

**Quantitative real-time PCR (qPCR) for *S. salivarius*, *S. parasanguinis* and LGG in the colon contents**

*S. salivarius*, *S. parasanguinis*, and LGG in the pup colon contents were quantified by qPCR on a LightCycler 96 system (Roche Applied Science, USA) using SYBR green I PCR Supermix (Bio–Rad, USA). The sequences of the primers and their annealing temperatures are listed in Supplementary Table S1. Each 20-μl reaction mixture contained 1× Supermix, 12.5 pmol of each primer, and 1 μl 1:5 dilution of extracted colon content DNA. The amplification programs consisted of one cycle of 95 °C for 3 min followed by 40 cycles of 95 °C for 20 s, the annealing temperature (Supplementary Table S1) for 30 s, 72 °C for 30 s, and the fluorescence reading for 5 s. Melting curve analysis was performed after amplification by slowly heating the samples at temperatures from 70 °C to 95 °C with continuous fluorescence monitoring to confirm the specificity of the PCR. The gene copy number of the bacteria in the samples was quantified using standard curves constructed from known concentrations, which ranged from 1×10^2^ to 1×10^9^ copies/μl, of plasmids that contained the quantified gene fragments cloned from *S. salivarius* F286, *S. parasanguinis* F278, and LGG, respectively, using the primer pairs listed in Supplementary Table S1. Each PCR was performed in triplicate.

**16S rRNA gene** **V3-V4 region sequencing data analysis**

The raw paired-end reads were processed with QIIME2 V2019.7. Adapters and primers were removed with q2-cutadapt. Trimming, denoising, merging and chimera detection were performed with “qiime dada2 denoise-paired” (2). Amplicon sequence variants (ASVs) were inserted into a rooted phylogenetic tree with fasttree2 via q2-phylogeny. The taxonomy of individual ASVs was determined with q2-feature-classifier. Alpha diversity indices, beta diversity distance matrices, and principal coordinate analysis (PCoA) were computed with q2-diversity. The significance of differences in colonic microbiota structure among the animal groups was assessed by permutational multivariate analysis of variance (PERMANOVA; 9,999 permutations) based on weighted UniFrac distances using the “vegan” R package, and differences were considered significant when p < 0.05. The centered and log_10_-transformed relative abundances of ASVs were used to construct sparse partial least-squares discriminant analysis (sPLS-DA) models (3) using the “mixOmics” R package, and ASVs with abundances that contributed to the discrimination of two animal groups were identified. The optimal classification performances of the sPLS-DA models were estimated by the perf function using leave-one-out cross-validation with the smallest balanced error rate. The relative abundances of differential ASVs among different groups identified by sPLS-DA were compared by the Mann–Whitney U test, and differences were considered significant when p < 0.05. Heatmaps showing the abundances of ASVs in animal groups were generated with the “pheatmap” R package.

**RT–qPCR for immune gene expression quantification of mice**

The integrity of the extracted RNA was checked by agarose gel electrophoresis, and the concentration was measured with a NanoVue spectrophotometer (GE Healthcare, Waukesha, WI, USA). One microgram of individual RNA samples was treated with DNase I (Invitrogen Life Technologies, USA) to remove contaminant DNA. Complementary DNA (cDNA) was generated from 500 ng of high-quality total RNA with random hexanucleotide primers and SuperScript III reverse transcriptase (Invitrogen, USA). qPCR was performed on a LightCycler 96 system (Roche, USA) using SYBR Green I PCR Supermix (Bio–Rad, USA) to quantify the mRNA expression levels of T-bet, IFN-γ, Gata3, IL-4, Rorγt, Foxp3, TGF-β, IL-10, Defβ1, and RegⅢγ. The sequences of the primers and their annealing temperatures are listed in Supplementary Table S1. The amplification programs consisted of one cycle of 95 °C for 3 min, followed by 40 cycles of 95 °C for 20 s, 56 °C for 30 s, and 72 °C for 30 s, and fluorescence reads for 5 s. The gene expression levels were determined using the ΔΔ*C_T_* method (2^-ΔΔ^*^CT^* method) with the *β-actin* gene as the reference gene.

**Bacterial coculture with human PBMCs**

Human PBMCs were isolated from the whole blood of these adults using Ficoll-Paque density gradient centrifugation (Ficoll-Paque, GE Healthcare) according to the manufacturer’s instructions, resuspended in RPMI 1640 (HyClone, CA, USA) supplemented with 2 mM L-glutamine, penicillin (100 IU/ml), streptomycin (100 μg/ml) and 10% fetal bovine serum (HyClone, CA, USA), and then seeded in 24-well plates (Corning) at 2 × 10^6^ cells/well.

In the heat-killed bacterial cell assays, 20 μl thawed bacterial cell suspension containing 1 × 10^8^ CFU/ml [MOI of 1, bacteria-to-cell ratio 1:1] or 1 × 10^9^ CFU/ml (MOI of 10, bacteria-to-cell ratio 10:1) and 20 μl PBS (nonstimulated negative control) was added to individual culture wells of PBMCs, and the final total culture volume was 1 ml. In the bacterial culture supernatant assays, to achieve 2% or 10% final concentrations, 20 μl and 100 μl bacterial culture supernatants, unfermented sterile bacteria medium M17 (control for *S. parasanguinis* F278 and *S. salivarius* F286) and MRS (control for LGG), respectively, were added to the individual culture wells of PBMCs, and the final total culture volume was 1 ml. Each assay was performed in triplicate. After 24 hours of stimulation at 37 °C in an atmosphere of air with 5% CO_2_, PBMC culture supernatants were collected, clarified by centrifugation at 300 ×g for 10 min, and stored at -80 °C until cytokine analysis. Cytokines were measured with ELISA kits for IL-10 (Invitrogen, CA, USA) and IL-12p70 (Biolegend, CA, USA) according to the manufacturer’s instructions.

**Bacterial coculture with TNF-α stimulated HT29 cells**

HT29 cells were cultured in Dulbecco’s modified Eagle’s medium (DMEM) (HyClone, CA, USA) supplemented with 10% fetal bovine serum (HyClone, CA, USA), penicillin (100 IU/ml) and streptomycin (100 μg/ml) at 37 °C in an atmosphere of air with 5% CO_2_. Cells were seeded into 24-well culture plates (Corning) at a density of 5 × 10^4^ cells/well in 1 ml DMEM. The medium was changed every second day. After culturing for 6 days, the HT29 cell culture medium was changed to DMEM culture medium with 5% fetal bovine serum, and cells were cultured for an additional 24 h. Monolayer-confluent HT29 cells (2×10^6^ cells/well) were rinsed with PBS (HyClone, CA, USA) once and then stimulated with human recombinant 5 ng/ml TNF-α (PeproTech, USA) in DMEM supplemented with heat-killed bacterial cells or bacterial culture supernatants. For the heat-killed bacterial cell assays, 50 µl thawed heat-killed bacterial cell suspension containing 1.6 × 10^9^ CFU/ml [MOI of 40, bacteria-to-cell ratio 40:1] or 4 × 10^9^ CFU/ml (MOI of 100, bacteria-to-cell ratio 100:1) and 50 μl PBS (nonstimulated negative control) was added to individual culture wells of HT29 cells, and the final total culture volume was 500 µl. In the bacterial culture supernatant assays, to achieve a final concentration of 10%, 50 μl bacterial culture supernatants, unfermented sterile bacteria medium M17 (negative control for *S. parasanguinis* F278 and *S. salivarius* F286) and MRS (negative control for LGG) were added to individual culture wells of HT29 cells, and the final total culture volume was 500 µl. After 6 hours of incubation, HT29 cell culture supernatant was collected, centrifuged at 4000 × g for 5 min and frozen at -20 °C until analysis. IL-8 was measured using Human IL-8/CXCL8 Quantikine ELISA kits (R&D Systems, MN, USA). Bacterial cells of *S. parasanguinis* F278, *S. salivarius* F286, and LGG at MOIs of 40 and 100 and 10% bacterial culture supernatants of the three strains did not affect the viability of HT-29 cells according to LDH assay (Roche Diagnostics, Mannheim, Germany).

**Quantifying the worm gene expression levels with RT–qPCR**

Five hundred worms fed individual bacterial strains from the L4 stage for 14 days were harvested and washed twice with sterilized M9 buffer. Total RNA was isolated from the worms using TRIzol reagent (Invitrogen, USA) according to the manufacturer’s instructions and purified using an RNeasy Mini Kit (QIAGEN, Hilden, Germany). Contaminant DNA was removed using a DNaseI kit (Invitrogen, USA), and DNA contamination was tested by PCR with primers targeting the housekeeping gene *act-1.* Total RNA was converted to complementary DNA (cDNA) using the SuperScript™ III First-Strand Synthesis System (Invitrogen Life Technologies, Carlsbad, CA, USA). Real-time quantitative PCR was performed with iQ^TM^ SYBR^®^ Green Surpermix (BIO-RAD, Hercules, CA, USA) on a LightCycler 96 machine (Roche, Geneva, Switzerland). The primer sequences are listed in Supplementary Table S2. The 2^−ΔΔCT^ method was used to quantify the mRNA level relative to that of *act*-1. The gene expression levels of *C. elegans* groups fed *S. parasanguinis* F278, *S. salivarius* F286, and LGG were expressed as values relative to the *E. coli* OP50-fed *C. elegans*.

**References**

1. Kaci G, Goudercourt D, Dennin V, Pot B, Dore J, Ehrlich SD, et al. Anti-inflammatory properties of Streptococcus salivarius, a commensal bacterium of the oral cavity and digestive tract. *Appl Environ Microbiol*. (2014) 80(3):928-34. doi: 10.1128/AEM.03133-13.

2. Callahan BJ, McMurdie PJ, Rosen MJ, Han AW, Johnson AJ, Holmes SP. DADA2: High-resolution sample inference from Illumina amplicon data. *Nat Methods*. (2016) 13(7):581-3. doi: 10.1038/nmeth.3869.

3. Le Cao KA, Boitard S, Besse P. Sparse PLS discriminant analysis: biologically relevant feature selection and graphical displays for multiclass problems. *BMC Bioinformatics*. (2011) 12:253. doi: 10.1186/1471-2105-12-253.
