# Supplementary material for: Gut Microbiota and Immune Modulatory Properties of Human Breast Milk Streptococcus salivarius and S. parasanguinis Strains
Source: Front Nutr. 2022 Feb 22;9:798403. doi: 10.3389/fnut.2022.798403 (PMC8901577; doi:10.3389/fnut.2022.798403)
Supplement: Supplementary file 3 [file Data_Sheet_3.pdf]

Gut Microbiota and Immune Modulatory Properties of Human Breast Milk *Streptococcus salivarius* and *S. parasanguinis* Strains

Supplementary Figures

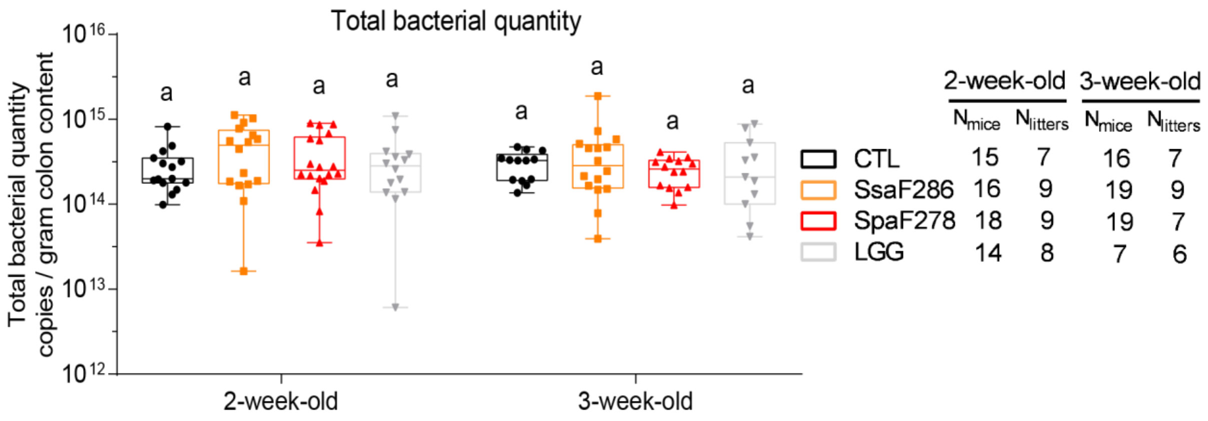

Supplementary Figure S1 Total amounts of colonic bacteria determined with qPCR as the total copy numbers of the 16S rRNA gene per gram of colonic contents. Oral feeding of *S. salivarius* F286, *S. parasanguinis* F278 or LGG did not change the total bacterial amounts in the colon contents of pups at the age of 2 and 3 weeks. These numbers were over evaluated due to PCR bias, however, samples from all mice, regardless the group and age, were quantified simultaneously in one PCR assay, so the overestimated bacterial quantity did not affect the comparison among groups or between ages. In the box plots, the bottom and top are respectively the 25th and 75th percentile, and the line within the boxes marks the median. Whiskers above and below the boxes indicate the minimal and maximal value respectively. Values of individual animal groups with identical letters are not significantly different by Mann-Whitney test. CTL, Control group; SsaF286, *S. salivarius* F286 group; SpaF278, *S. parasanguinis* F278 group; LGG, LGG group.

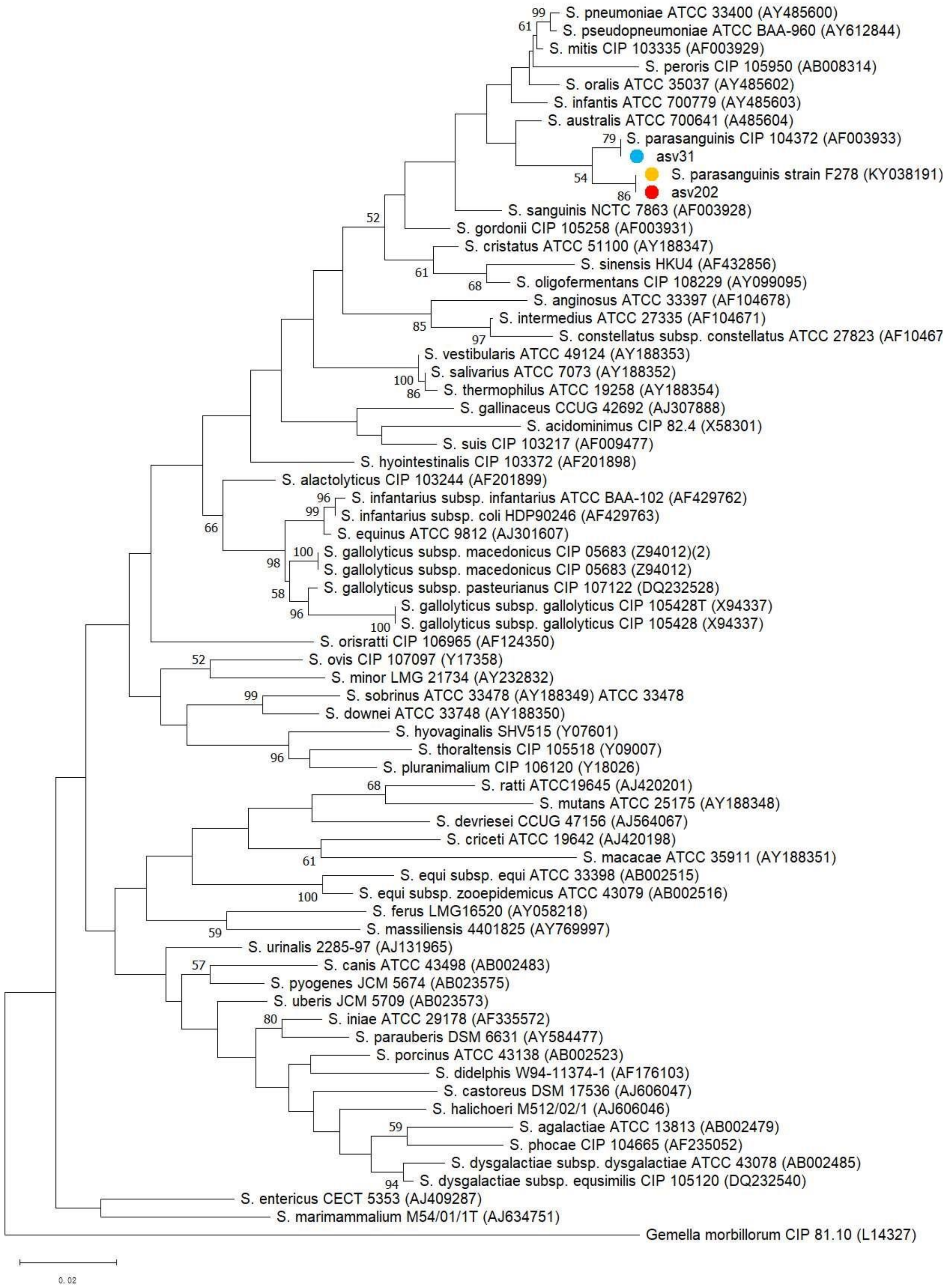

Supplementary Figure S2 Phylogenetic tree showing that ASV202 and ASV31 were unambiguously clustered with *S. parasanguinis*. The maximum likelihood phylogenetic tree contained the 16S rRNA gene sequences of type strains of *Streptococcus* spp. (see reference Glazunova, O. O., Raoult, D., and Roux, V. 2009. Partial sequence comparison of the rpoB, sodA, groEL and gyrB genes within the genus Streptococcus. Int. J. Syst. Evol. Microbiol. 59(Pt 9), 2317–2322. doi: 10.1099/ijs.0.005488-5480) and *S. parasanguinis* F278 (labeled by a yellow dot), and the sequences of ASV202 (labeled by a red dot) and ASV31 (labeled by a blue dot). The phylogenetic tree was constructed with the Molecular Evolutionary Genetics Analysis package 7 (MEGA 7). The phylogenetic robustness was assessed by bootstrap analysis with 1000 replicates. Bootstrap values greater than 50% are indicated at the nodes. The sequence of *Gemella morbillorum* CIP 81.10T was used as the outgroup.

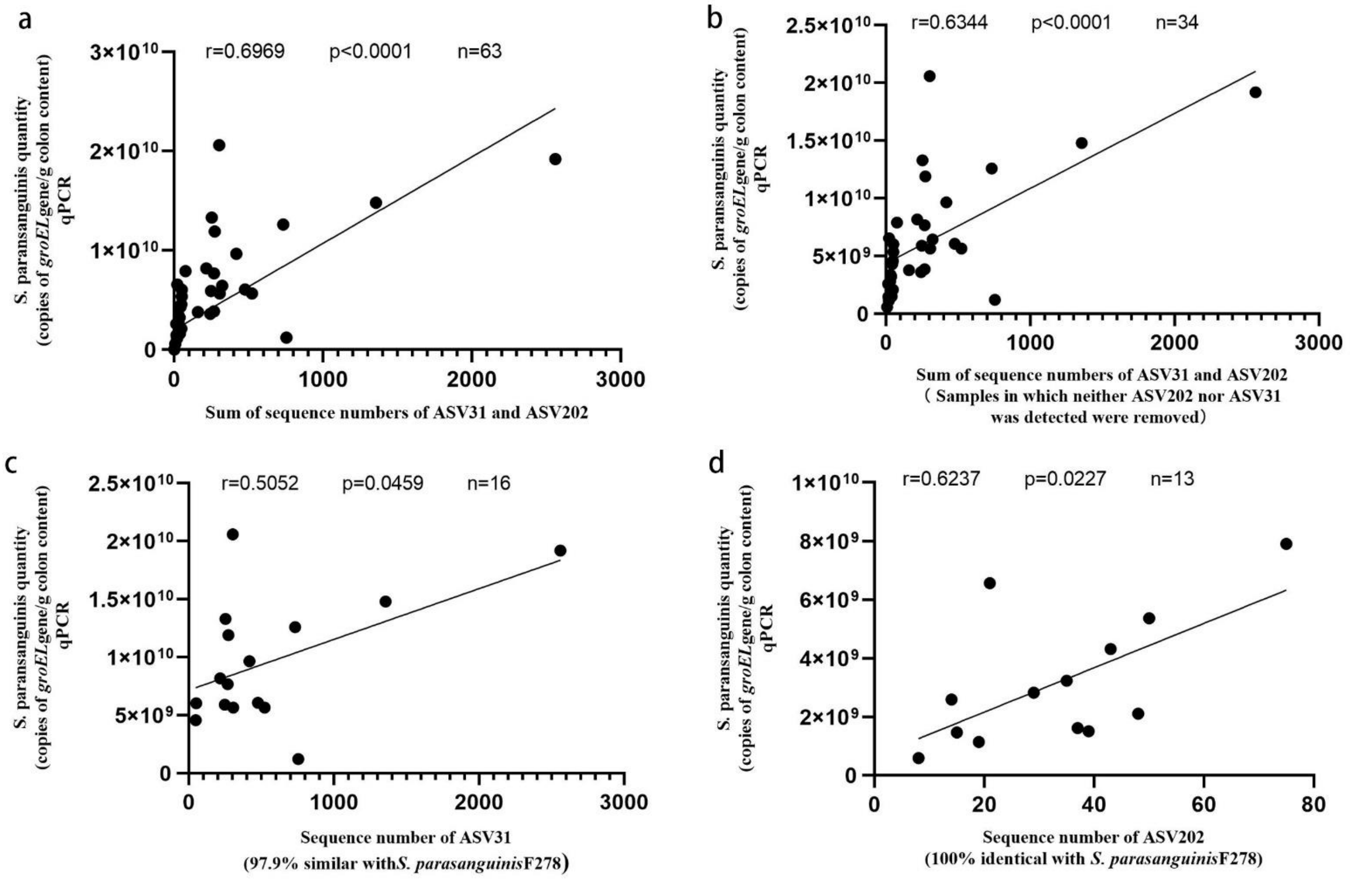

Supplementary Figure S3 Bivariate scatterplots of the Pearson's correlation of sequence numbers of two *S. parasanguinis* ASVs (ASV31 and ASV202) and the amounts of *S. paransanguinis* as copies of *groEL* gene /g colon content determined with qPCR in 2-week-old mice. The correlation coefficient (r), p value, and the number of colon content samples (n) are shown in the plots. The data of ASV sequence numbers and qPCR of individual mice were shown in Supplementary Table S4. (a) In all mice no matter the two ASVs were detected or not, the sum of sequence numbers of ASV31 and ASV202 was significantly correlated with the *S. paransanguinis* amount determined with qPCR. (b) In mice in which either ASV31 or ASV202 was detected, the sum of sequence numbers of ASV31 and ASV202 was significantly correlated with the amount of *S. paransanguinis* determined with qPCR. (c) In mice in which only ASV31 was detected, the number of ASV31 was significantly correlated with the *S. paransanguinis* abundance determined with qPCR. (d) In mice in which only ASV202 was detected, the number of ASV202 was significantly correlated with the *S. paransanguinis* abundance determined with qPCR.

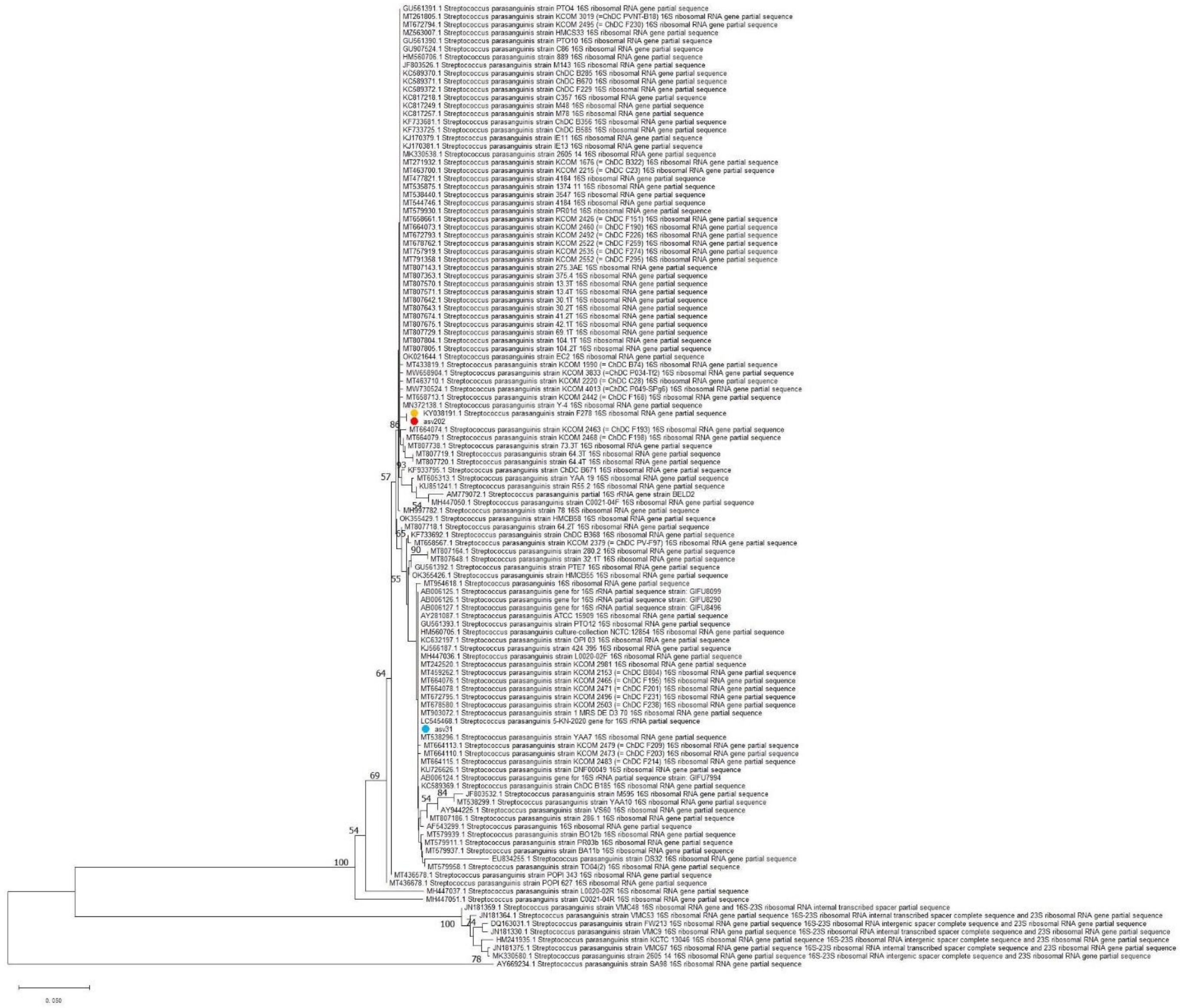

Supplementary Figure S4 Phylogenetic tree showing that ASV202 and ASV31 distributed in different clusters of *S. parasanguinis* strains. The tree contained the 16S rRNA gene sequences of 115 *S. parasanguinis* strains which were downloaded from the Genbank of NCBI, and the sequences of ASV202 (labeled by a red dot) and ASV31 (labeled by a blue dot). The phylogenetic tree was constructed with the Molecular Evolutionary Genetics Analysis package 7 (MEGA 7). The phylogenetic robustness was assessed by bootstrap analysis with 1000 replicates. Bootstrap values greater than 50% are indicated at the nodes.

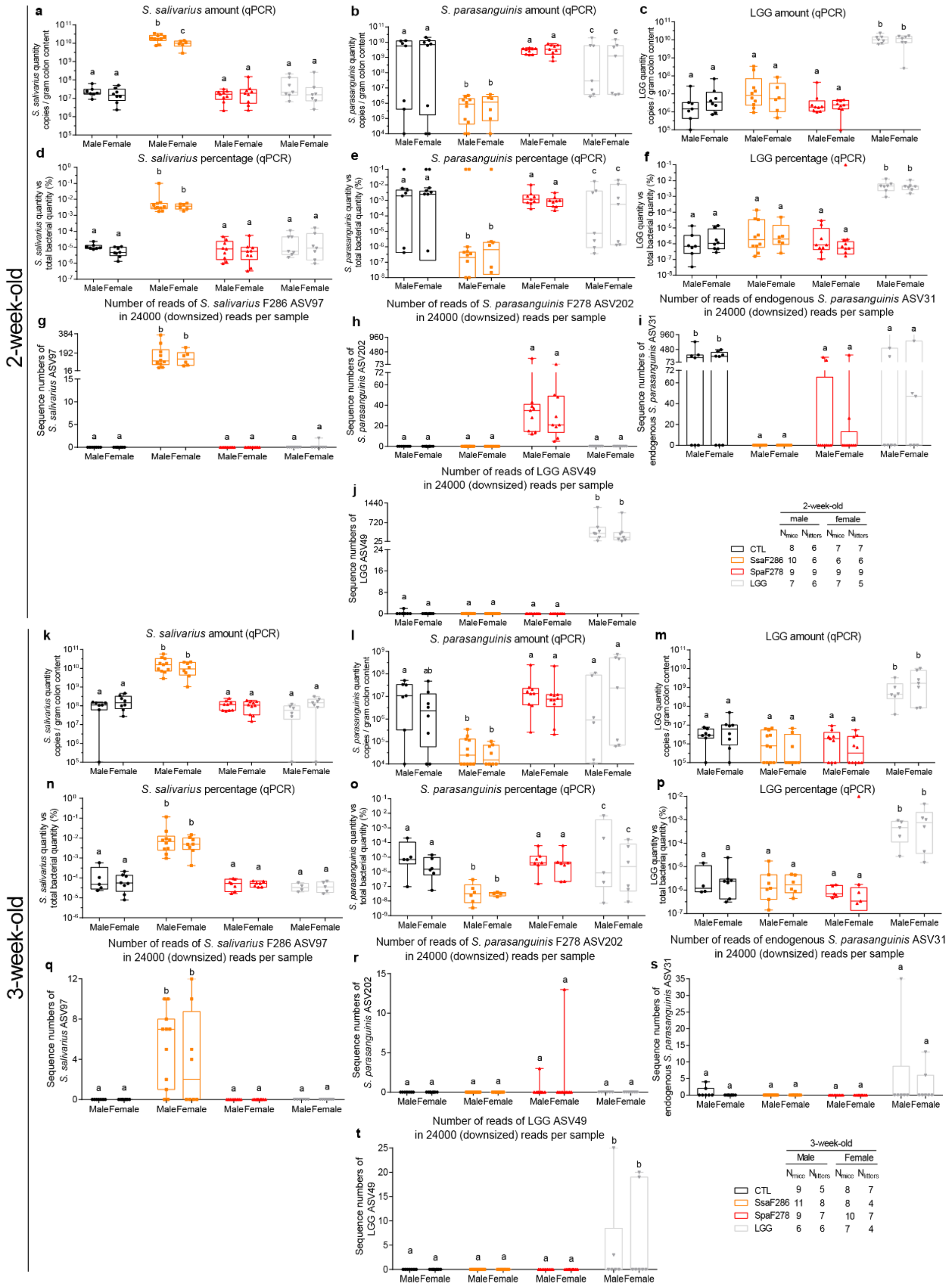

Supplementary Figure S5 The male and female pups did not show differences in the abundance of colonic S. salivarius F286, S. parasanguinis F278 and LGG at the ages of 2 and 3 weeks. (a-c) The amounts of *S. salivarius*, *S. paransanguinis* and LGG determined with qPCR as the copy numbers of the targeted gene of individual bacteria per gram of colon contents of 2- and 3-week-old mouse pups. (d-f) Percentages of copy numbers of the targeted genes of *S. salivarius*, *S. paransanguinis* and LGG accounted to the total copy numbers of 16S rRNA gene (Supplementary Figure S1) in the colon contents of pups. (g-h) Sequencing read numbers of 16S rRNA gene V3-V4 region ASVs identified as *S. salivarius* F286, *S. paransanguinis* F278, the endogenous *S. paransanguinis* strain and LGG in the colon contents of pups. In the box plots, the bottom and top are respectively the 25th and 75th percentile, the line within the box marks the median. Whiskers above and below the box indicate the minimal and maximal value respectively. Values of individual animal groups with identical letters are not significantly different by Mann-Whitney test. CTL, Control group; SsaF286, *S. salivarius* F286 group; SpaF278, *S. parasanguinis* F278 group; LGG, LGG group.

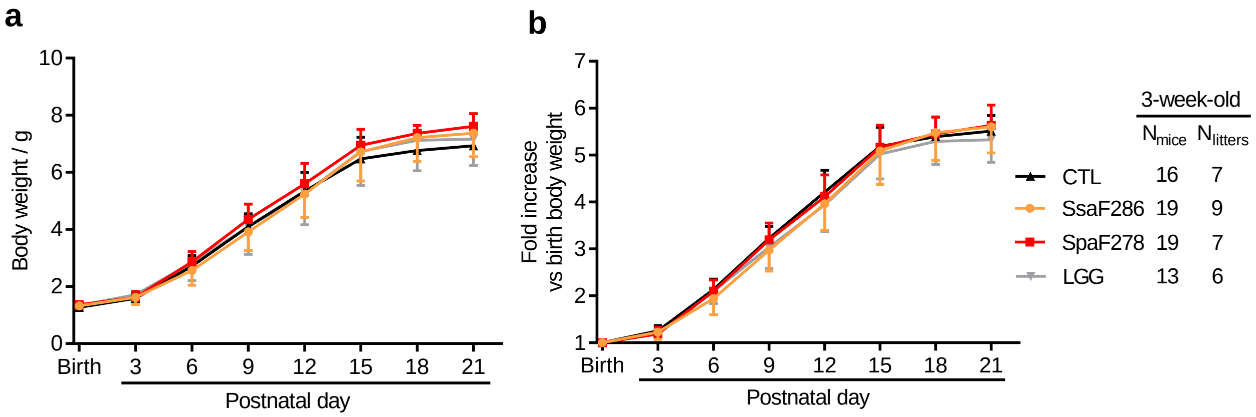

Supplementary Figure S6 *S. salivarius* F286, *S. parasanguinis* F278 and LGG did not affect the body weight or body weight gain of neonatal mice in the first 3 weeks of life. (a) body weight curves. (b) Body weight gain as the fold increase relative to the birth weight. Birth weight is set as 1. Values are presented as mean ± SD. The data of *S. salivarius* F286-, *S. parasanguinis* F278- and LGG-fed animals were compared to those of control animals with Student's t test at each time points, and showed no significant difference. CTL, Control group; SsaF286, *S. salivarius* F286 group; SpaF278, *S. parasanguinis* F278 group; LGG, LGG group.

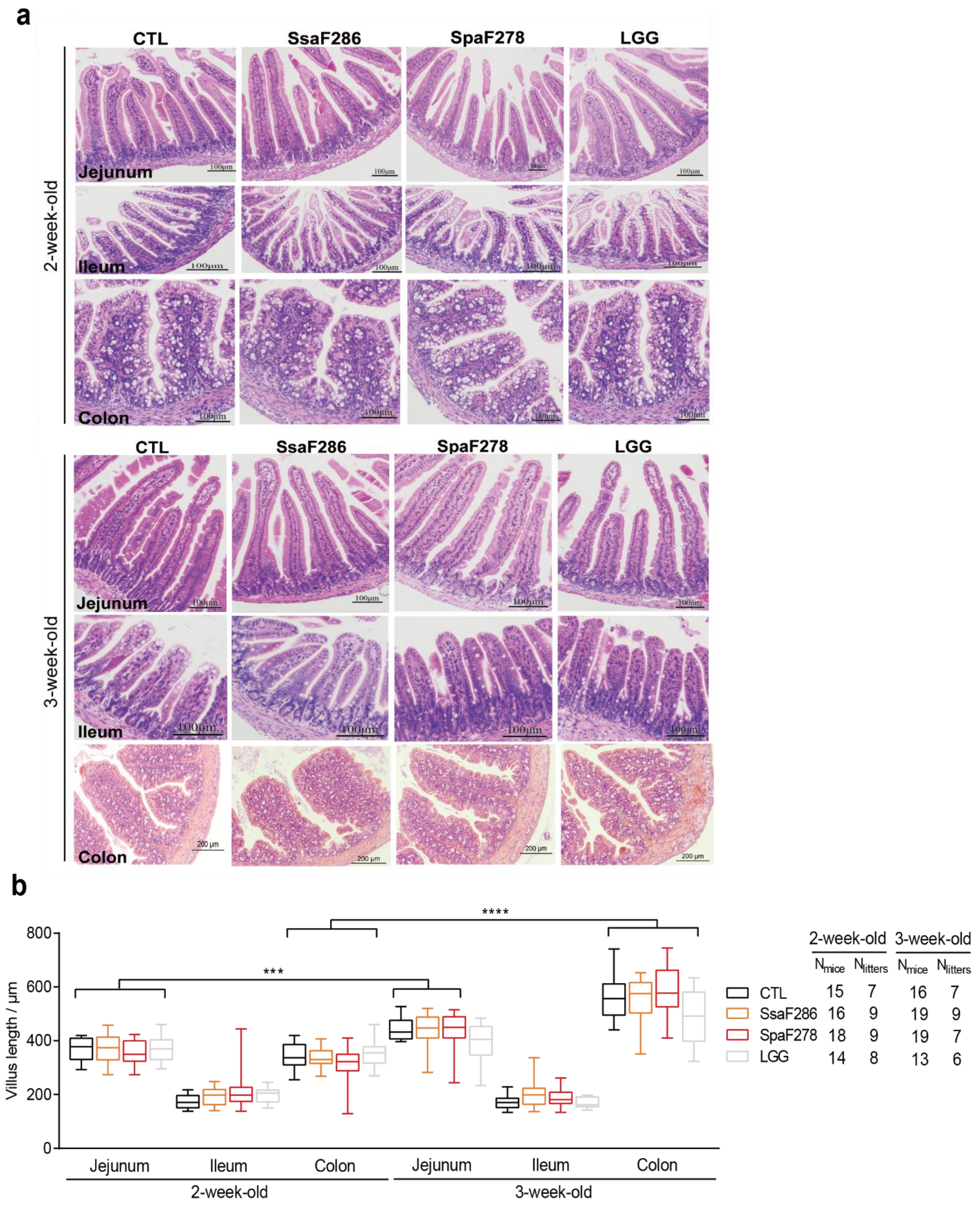

Supplementary Figure S7 *S. salivarius* F286, *S. parasanguinis* F278, and LGG did not affect intestinal villus length. (a) Hematoxylin and eosin-stained sections of jejunum, ileum and colon of 2-week-old and 3-week-old pups. (b)Villi length of jejunum, ileum and colon in each animal group measured in at least 30 villi/mouse at the age of week 2 and 3. In the box plot, the bottom and top are respectively the 25th and 75th percentile, a line within the box marks the median. Whiskers above and below the box indicate as minimal and maximal value. *P*-values were determined by Mann-Whitney test. *, $p < 0.05$; **, $p < 0.01$; ***, $p < 0.001$; ****, $p<0.0001$. CTL, Control group; SsaF286, *S. salivarius* F286 group; SpaF278, *S. parasanguinis* F278 group; LGG, LGG group.

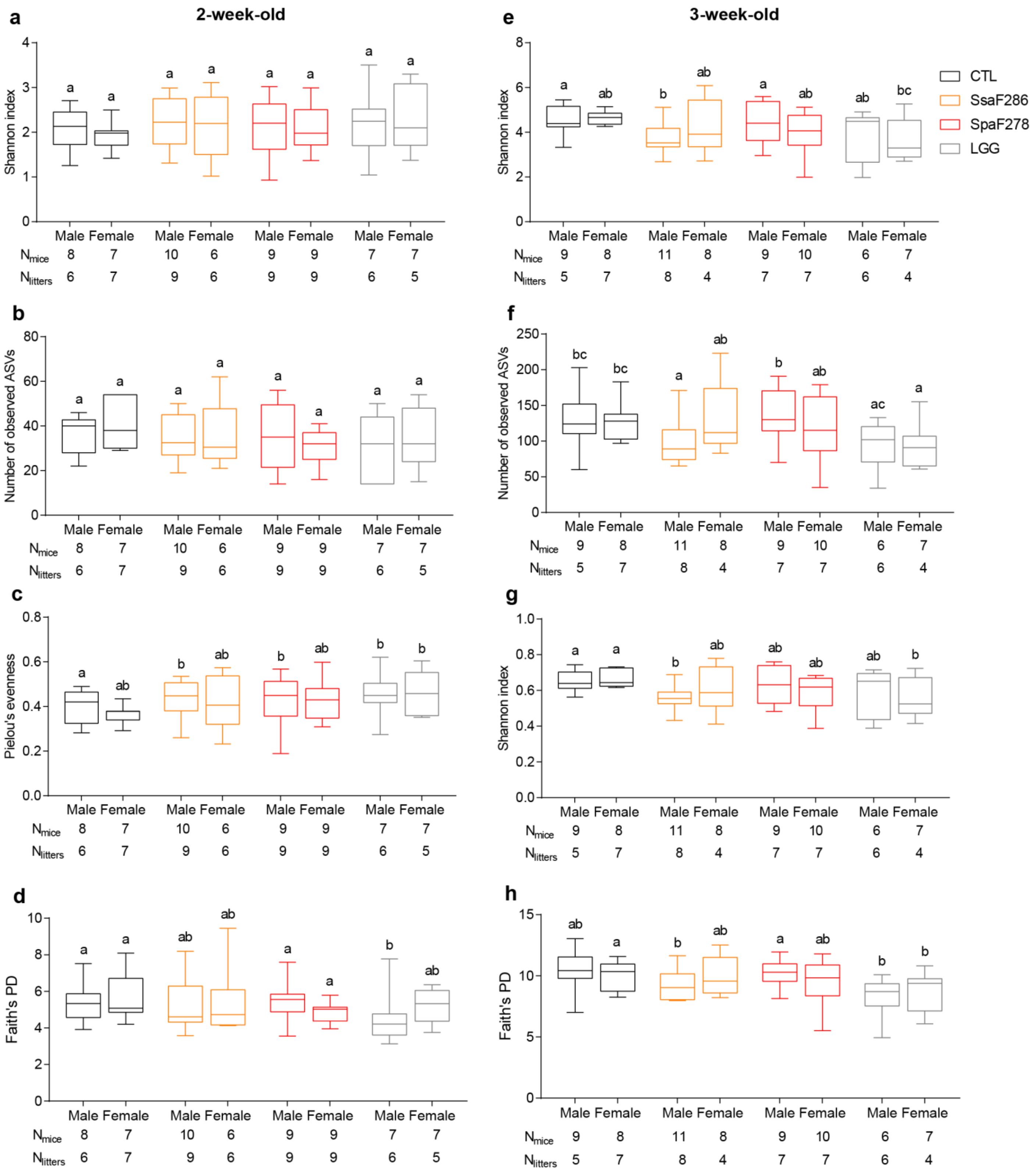

Supplementary Figure S8 The male and female pups did not show differences in the alpha diversity of the colonic microbiota at the ages of 2 (a-d) and 3 (e-h) weeks. In the box plots, the bottom and top are respectively the 25th and 75th percentile, the line within the box marks the median. Whiskers above and below the box indicate as minimal and maximal value. Values of individual animal groups with identical letters are not significantly different by Mann-Whitney test. CTL, Control group; SsaF286, *S. salivarius* F286 group; SpaF278, *S. parasanguinis* F278 group; LGG, LGG group.

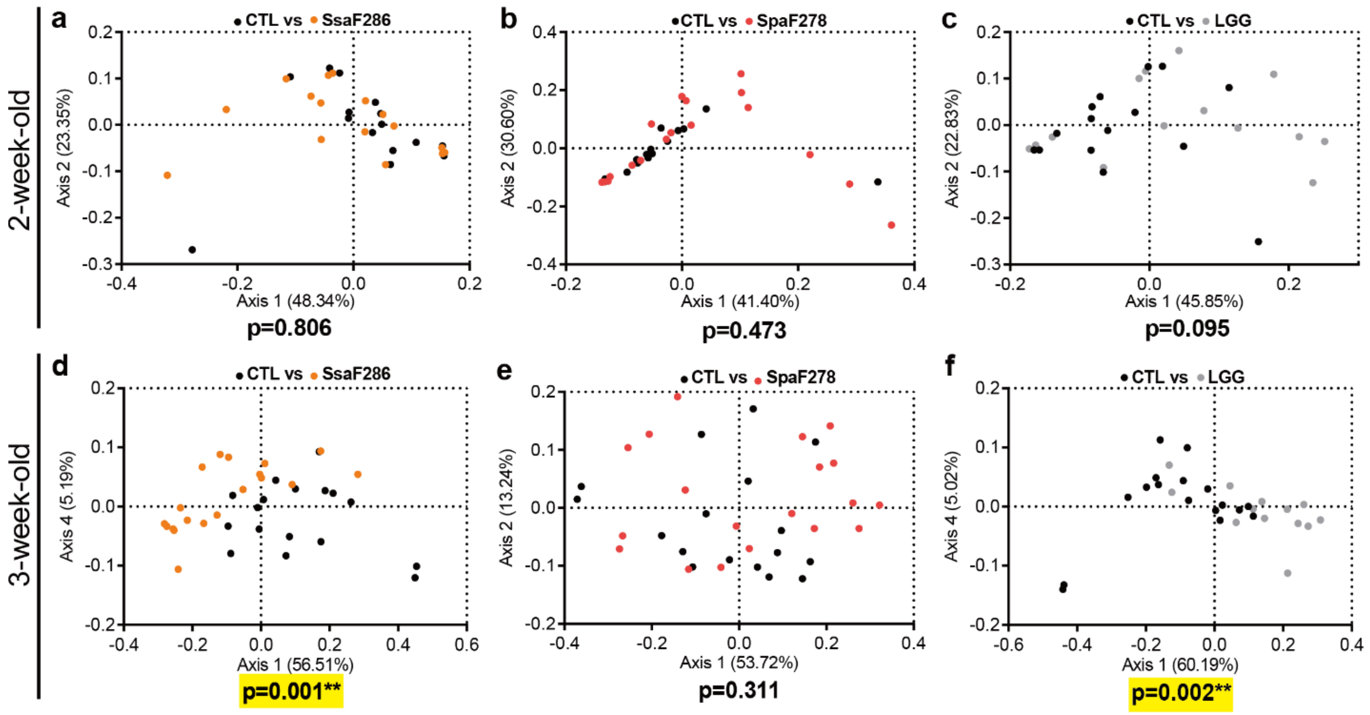

Supplementary Figure S9 *S. salivarius* F286 and LGG changed the overall structure of the colonic microbiota of 3-week-old pups. PCoA plots based on weighted UniFrac distance of colonic microbiota comparing pups fed individual bacterial strains to control pups at the age of 2 weeks (a-c) and 3 weeks (d-f). p values of permutational multivariate analysis of variance (PERMANOVA; 9,999 permutations) between two animal groups are shown at the bottom of the plots, and p values that less than 0.05 are highlighted. CTL, Control group; SsaF286, *S. salivarius F286* group; SpaF278, *S. parasanguinis* F278 group; LGG, LGG group.

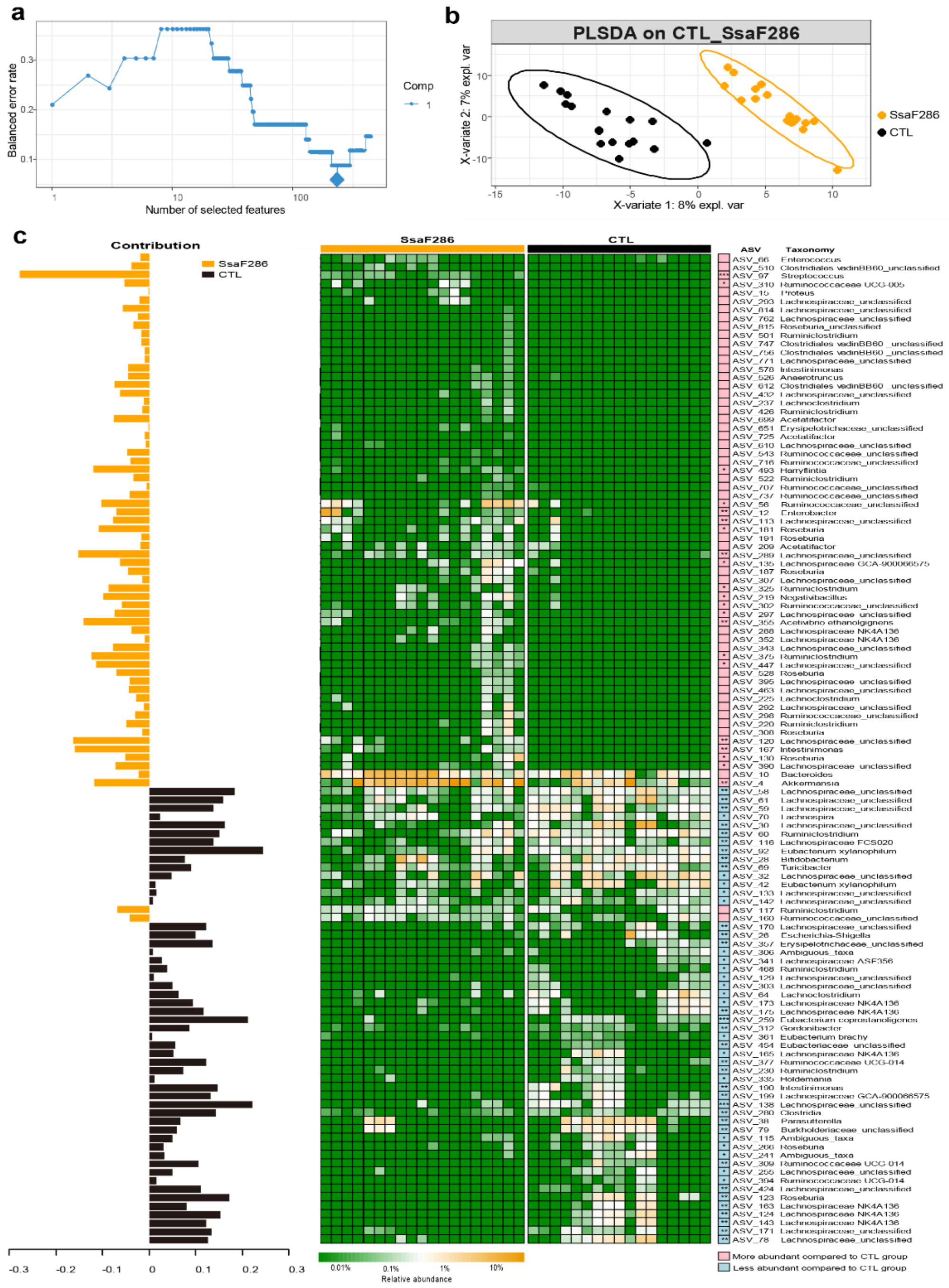

Supplementary Figure S10 The sPLS-DA model for the separation of colonic microbiota of *S. salivarius* F286-fed and control pups at the age of 3 weeks and the ASVs whose abundance were changed by *S. salivarius* F286. (a) The classification error rate of the component of the sPLS-DA model according to the leave-one-out cross-validation. (b) The sPLS-DA plots of gut microbiota of *S. salivarius* F286-fed and control pups at the age of 3 weeks based on ASV table. (c) 117 ASVs were identified to contribute to the separation of two animal groups by the sPLS-DA model, and 73 of them were significantly different between two animal groups according to Mann-Whitney U test. Left panel, contribution plot of the ASVs showing the contribution of each ASV to the discrimination of two animal groups in the sPLS-DA model. Middle panel, heatmap of the normalized and $\log_{10}$-transformed relative abundance of 117 ASVs. Right panel, the changing direction of 117 ASVs in *S. salivarius* F286-fed animals compared to control counterparts, and the significance of their abundance difference between two animal groups in Mann-Whitney U test. Redness and blueness indicate the relative abundance of ASVs were more and less, respectively, in *S. salivarius* F286 group compared to control group. * represents the relative abundance of the ASVs were significantly different between two animal groups in Mann-Whitney U test. *, $p < 0.05$; **, $p < 0.01$; ***, $p < 0.001$. The taxonomy of each ASV is shown. ASVs that were identified as differential ASVs by sPLS-DA and also significantly different in Mann-Whitney U test are considered to be significantly changed by *S. salivarius* F286.

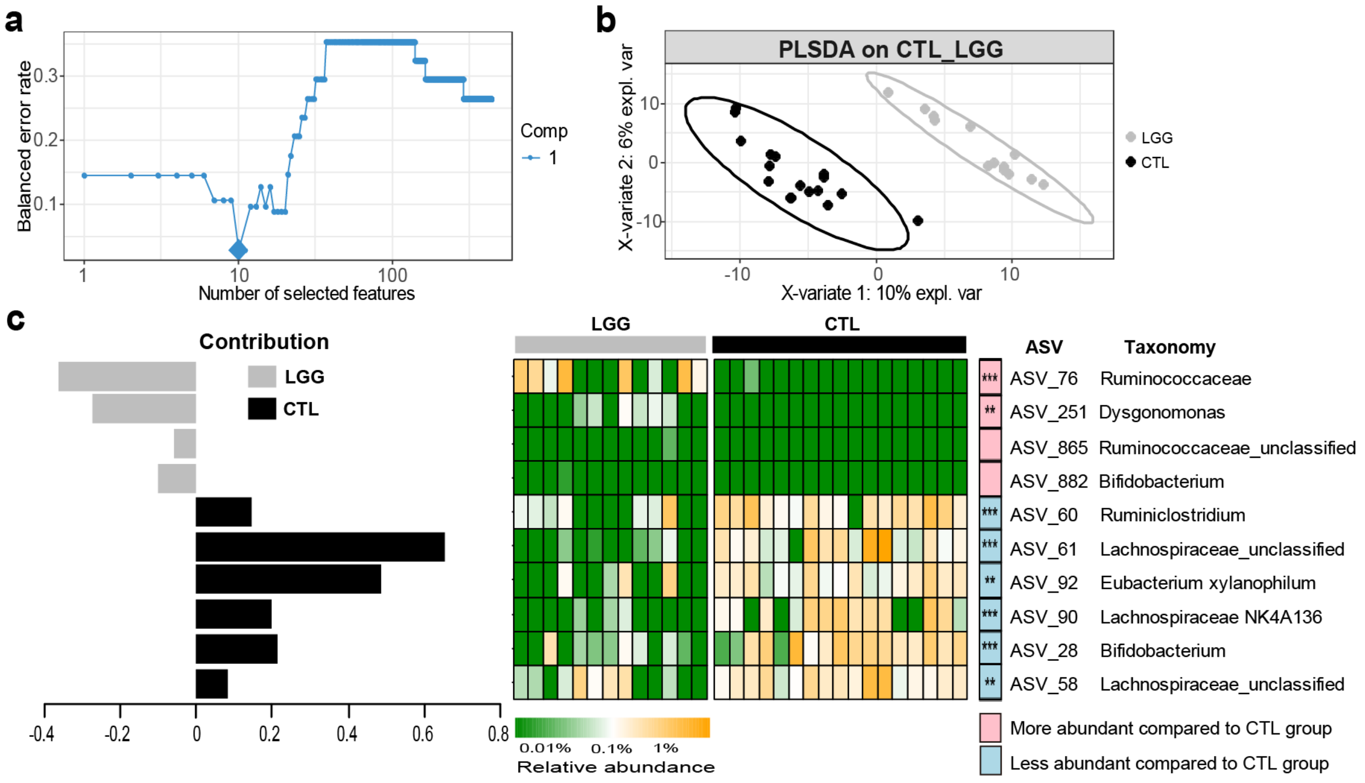

Supplementary Figure S11 The sPLS-DA model for the separation of colonic microbiota of LGG-fed and control pups at the age of 3 weeks and the ASVs whose abundance were changed by LGG. (a) The classification error rate of the component of the sPLS-DA model according to the leave-one-out cross-validation. (b) The sPLS-DA plots of gut microbiota of LGG-fed and control pups at the age of 3 weeks based on ASV table. (c) 10 ASVs were identified to contribute to the separation of two animal groups by the sPLS-DA model, and 8 of them were significantly different between two animal groups according to Mann-Whitney U test. Left panel, contribution plot of the 10 ASVs showing the contribution of each ASV to the discrimination of two animal groups in the sPLS-DA model. Middle panel, heatmap of the normalized and log_{10}-transformed relative abundance of 10 ASVs. Right panel, the changing direction of 10 ASVs in LGG-fed animals compared to control counterparts, and the significance of their abundance difference between two animal groups in Mann-Whitney U test. Redness and blueness indicate the relative abundance of ASVs were more and less, respectively, in LGG group compared to control group. * represents the relative abundance of the ASVs were significantly different between two animal groups in Mann-Whitney U test. *, $p < 0.05$; **, $p < 0.01$; ***, $p < 0.001$. The taxonomy of each ASV is shown. ASVs that were identified as differential ASVs by sPLS-DA and also significantly different in Mann-Whitney U test are considered to be significantly changed by LGG.

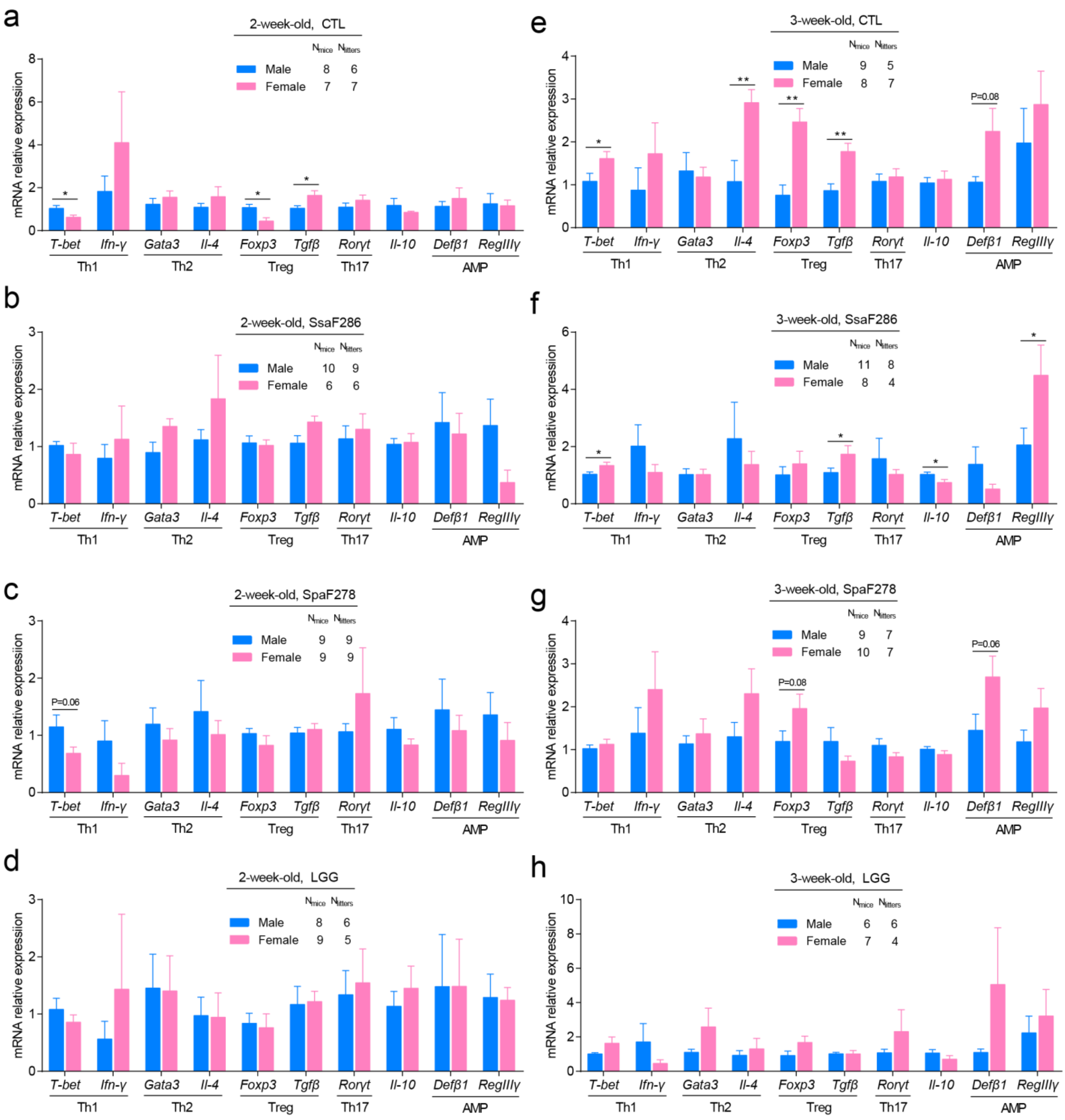

Supplementary Figure S12 The male and female pups showed gender difference in the expression levels of ileal immune genes at the age of 2 (a-d) and 3 weeks (e-h). All mRNA quantification data were normalized to the housekeeping gene *β-actin*. Gene expression levels were expressed as values relative to the male pups. Data are presented as mean ± s.e.m. For each animal group, the data of individual genes were compared between two genders with Student's t test. *, $p < 0.05$; **, $p < 0.01$. AMP, anti-microbial peptides. CTL, Control group; SsaF286, *S. salivarius* F286 group; SpaF278, *S. parasanguinis* F278 group; LGG, LGG group
